# Within-Host Genotypic and Phenotypic Diversity of Contemporaneous Carbapenem-Resistant Klebsiella pneumoniae from Blood Cultures of Patients with Bacteremia

**DOI:** 10.1128/mbio.02906-22

**Published:** 2022-11-29

**Authors:** Shaoji Cheng, Giuseppe Fleres, Liang Chen, Guojun Liu, Binghua Hao, Anthony Newbrough, Eileen Driscoll, Ryan K. Shields, Kevin M. Squires, Ting-yu Chu, Barry N. Kreiswirth, M. Hong Nguyen, Cornelius J. Clancy

**Affiliations:** a University of Pittsburgh grid.471408 grid.21925 , Pittsburgh, Pennsylvania, USA; b Hackensack Meridian Health Center for Discovery and Innovation, Nutley, New Jersey, USA; c University of Pittsburgh grid.471408 grid.21925 Medical Center, Pittsburgh, Pennsylvania, USA; d VA Pittsburgh Healthcare System, Pittsburgh, Pennsylvania, USA; McMaster University

**Keywords:** *Klebsiella pneumoniae*, sequence type 258, carbapenem resistance, within-host diversity, bloodstream infection, blood culture, whole-genome sequence

## Abstract

It is unknown whether bacterial bloodstream infections (BSIs) are commonly caused by single organisms or mixed microbial populations. We hypothesized that contemporaneous carbapenem-resistant Klebsiella pneumoniae (CRKP) strains from blood cultures of individual patients are genetically and phenotypically distinct. We determined short-read whole-genome sequences of 10 sequence type 258 (ST258) CRKP strains from blood cultures in each of 6 patients (Illumina HiSeq). Strains clustered by patient by core genome and pan-genome phylogeny. In 5 patients, there was within-host strain diversity by gene mutations, presence/absence of antibiotic resistance or virulence genes, and/or plasmid content. Accessory gene phylogeny revealed strain diversity in all 6 patients. Strains from 3 patients underwent long-read sequencing for genome completion (Oxford Nanopore) and phenotypic testing. Genetically distinct strains within individuals exhibited significant differences in carbapenem and other antibiotic responses, capsular polysaccharide (CPS) production, mucoviscosity, and/or serum killing. In 2 patients, strains differed significantly in virulence during mouse BSIs. Genetic or phenotypic diversity was not observed among strains recovered from blood culture bottles seeded with index strains from the 3 patients and incubated *in vitro* at 37°C. In conclusion, we identified genotypic and phenotypic variant ST258 CRKP strains from blood cultures of individual patients with BSIs, which were not detected by the clinical laboratory or in seeded blood cultures. The data suggest a new paradigm of CRKP population diversity during BSIs, at least in some patients. If validated for BSIs caused by other bacteria, within-host microbial diversity may have implications for medical, microbiology, and infection prevention practices and for understanding antibiotic resistance and pathogenesis.

## INTRODUCTION

Carbapenem-resistant *Enterobacterales* (CRE) are “urgent threat” pathogens globally (1–3). Klebsiella pneumoniae (CRKP) is the most common CRE worldwide (4, 5). Most CRE infections are caused by commensal strains from the gastrointestinal (GI) tract (6–8). Recent whole-genome sequencing (WGS) data demonstrate that colonization or chronic infections by various bacteria may be caused by a population of clonal strains, in which genetic diversity emerges during long-term host interactions (9–22). Such clonal but genetically diverse strains can manifest distinct phenotypes that are potentially relevant to commensalism or persistence in the host (9, 10). At present, it is unknown how commonly acute monomicrobial infections of putatively sterile sites are caused by genetically and phenotypically diverse bacterial populations.

Prompt and accurate diagnosis of bacterial bloodstream infections (BSIs) is among the most critical functions of clinical microbiology laboratories (23). In processing positive microbiologic cultures, the standard practice is to isolate a strain from each morphologically distinct colony. This practice is in keeping with the long-standing model for the pathogenesis of bacteremia, in which most monomicrobial infections are believed to be due to a single organism (single-organism or independent-action hypothesis) (24–29). Approximately 10 to 15% of BSIs are polymicrobial, with more than one species recovered from positive blood cultures (30). A smaller percentage of BSIs are monomicrobial but polyclonal, caused by strains of the same species that differ by multilocus sequence type (ST) or pulsed-field gel electrophoresis patterns (31, 32). In this study, we tested the hypothesis that contemporaneous CRKP strains from individual patients diagnosed with monomicrobial, clonal BSIs are genetically and phenotypically diverse. We identified 6 patients with BSIs caused by ST258 CRKP (the predominant international clone). For each patient, the clinical laboratory isolated an index strain from a single colony morphotype. We analyzed WGSs of index and 9 other strains recovered from independent, morphologically indistinguishable colonies. We then assessed phenotypes of genetically distinct strains from 3 patients. Finally, we seeded sterile blood culture bottles with index strains from the 3 patients and determined if genetic and phenotypic variant strains emerged following growth *in vitro*.

## RESULTS

### Patients with CRKP BSIs.

We enrolled 6 adults (4 men, 2 women) who were diagnosed with monomicrobial CRKP BSIs (Table 1). Patients ranged from 32 to 76 years of age. Each patient had a complicated medical history, with serious underlying diseases and prior receipt of broad-spectrum antibiotics. Five patients had diseases and/or surgical interventions involving the GI tract; five patients had had at least one hospitalization within the preceding 6 months. Four patients had had previous invasive infections caused by CRKP. In two patients, BSIs were complicated by concurrent CRKP pneumonia. Likely portals of entry for BSI were the GI tract (*n* = 5) and an intravenous catheter (*n* = 1). Two patients died within 30 days of BSI diagnosis.

**TABLE 1:** Clinical characteristics of patients with carbapenem-resistant K. pneumoniae bloodstream infections^*a*^

Pt	Age (yrs), sex	Underlying conditions	Antibiotics (preceding 6 mo)	Hospitalization and CRKP infection (preceding 6 mo)	Concurrent CRKP infection	Likely BSI source	BSI treatment	Outcome (30 days)
A	67, M	Esophageal cancer, s/p esophagectomy and partial gastrectomy, colon interposition, open laparotomy and sternotomy	Multiple, including DOX, TZP, MEM	No recent hospitalization; intra-abdominal (polymicrobial including CRKP)	Pneumonia	GI	CZA, inhaled GEN	Alive
B	32, M	IVDU, MSSA endocarditis with septic emboli to multiple organs, s/p pneumonectomy, recurrent VAP, renal failure requiring hemodialysis	Multiple, including TZP, FEP, MEM, CZA, CST	Hospitalization 4 mo prior; pneumonia	NA	Line	CZA	Dead
D	52, M	Renal transplant, esophageal cancer, s/p esophagectomy and partial gastrectomy, substernal gastric pull-up, hemodialysis, GCV-R CMV	Multiple, including TZP, MEM, CIP	Hospitalization 4.5 mo prior; BSI, pneumonia, urine colonization	Pneumonia	GI	CZA, GEN	Alive
F	60, F	Enteric fistula, s/p small bowel resection, gastrostomy and jejunostomy, anastomotic leaks	Multiple, including TZP, FEP, CLI	3 hospitalizations (3, 4, and 5 mo prior); urine colonization	NA	GI	CZA	Alive
G	67, F	Hypertension, COPD, biliary stent occlusion, s/p ERCP and stent replacement	FEP, CIP	2 hospitalizations (2 and 3 mo prior); cholangitis, sepsis	Urine colonization	GI	CZA, i.v. GEN	Dead
J	76, M	Esophageal-pleural fistula, s/p ERCP and multiple stents, respiratory failure, recurrent VAP	Multiple, including TZP, MEM	Hospitalization 1 mo prior; no prior CRKP infection	NA	GI	MVB	Alive

a Abbreviations: Pt, patient; CRKP, carbapenem-resistant K. pneumoniae; s/p, status post-; GI, gastrointestinal; IVDU, intravenous drug user; MSSA, methicillin-susceptible Staphylococcus aureus; VAP, ventilator-associated pneumonia; GCV-R CMV, infection with ganciclovir-resistant cytomegalovirus; COPD, chronic obstructive pulmonary disease; ERCP, endoscopic retrograde cholangiopancreatography; NA, not applicable; i.v., intravenous; CZA, ceftazidime-avibactam; CIP, ciprofloxacin; CLI, clindamycin; CST, colistin; DOX, doxycycline; FEP, cefepime; GEN, gentamicin; MEM, meropenem; TZP, piperacillin-tazobactam.

### WGSs of CRKP from positive blood cultures.

We obtained index BSI strains isolated by the clinical microbiology laboratory from the 6 patients. We streaked aliquots from positive culture bottles from each patient onto blood agar plates and selected 9 colonies at random. We determined short-read WGSs of the index strain (labeled strain 1) and other strains (strains 2 to 10) (Illumina HiSeq). All strains were identified as ST258 K. pneumoniae. We submitted raw reads to the National Center for Biotechnology Information (NCBI) Sequence Read Archive (BioProject PRJNA826066). Information on genome assemblies is provided in Table S1 in the supplemental material. K. pneumoniae 30660/NJST258_1 served as reference genome for WGS analyses.

10.1128/mbio.02906-22.1 TABLE S1 Assembly data for carbapenem-resistant K. pneumoniae strains. Download Table S1, DOCX file, 0.02 MB. Copyright © 2022 Cheng et al. 2022 Cheng et al. https://creativecommons.org/licenses/by/4.0/ This content is distributed under the terms of the Creative Commons Attribution 4.0 International license.

**FIG 1:**
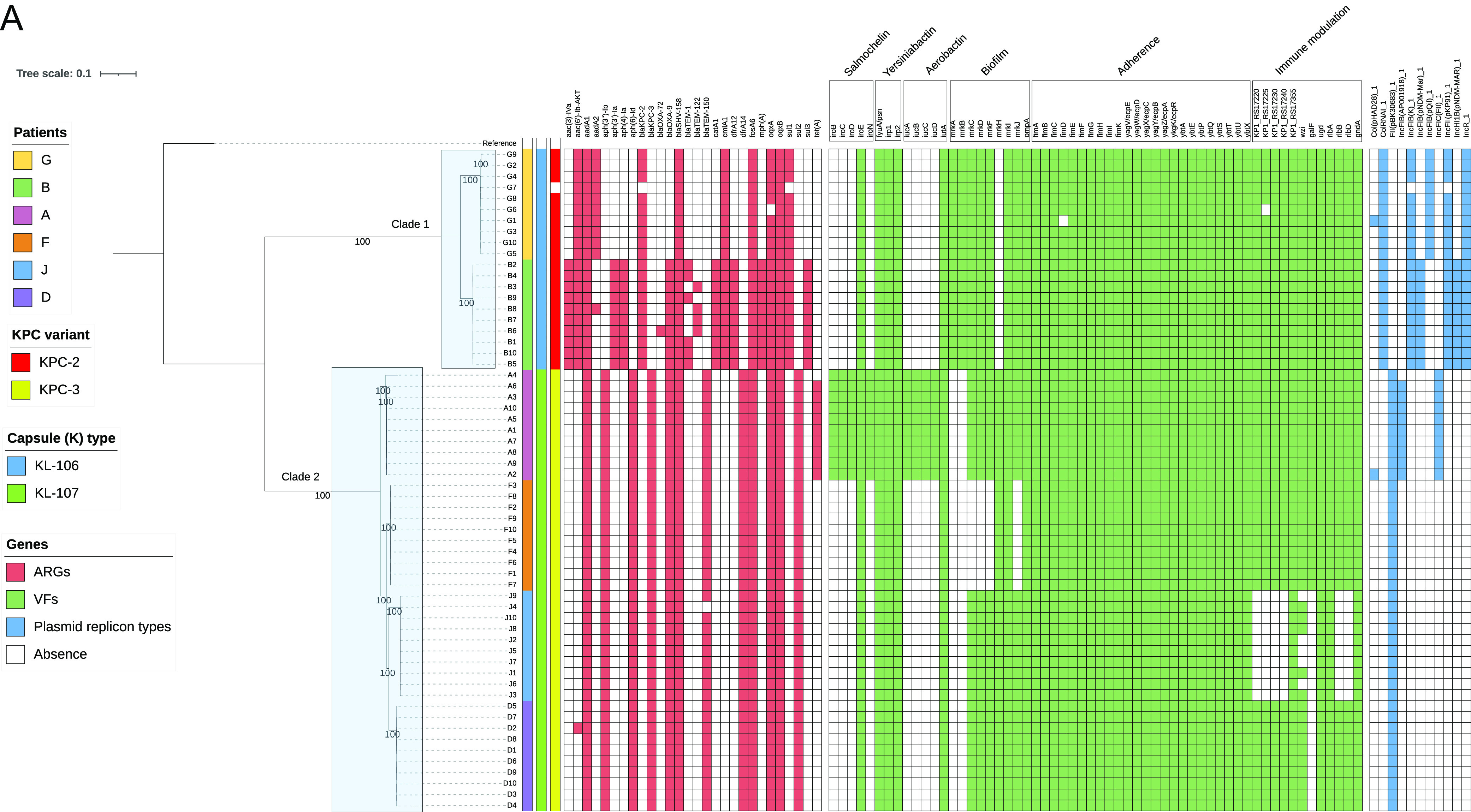

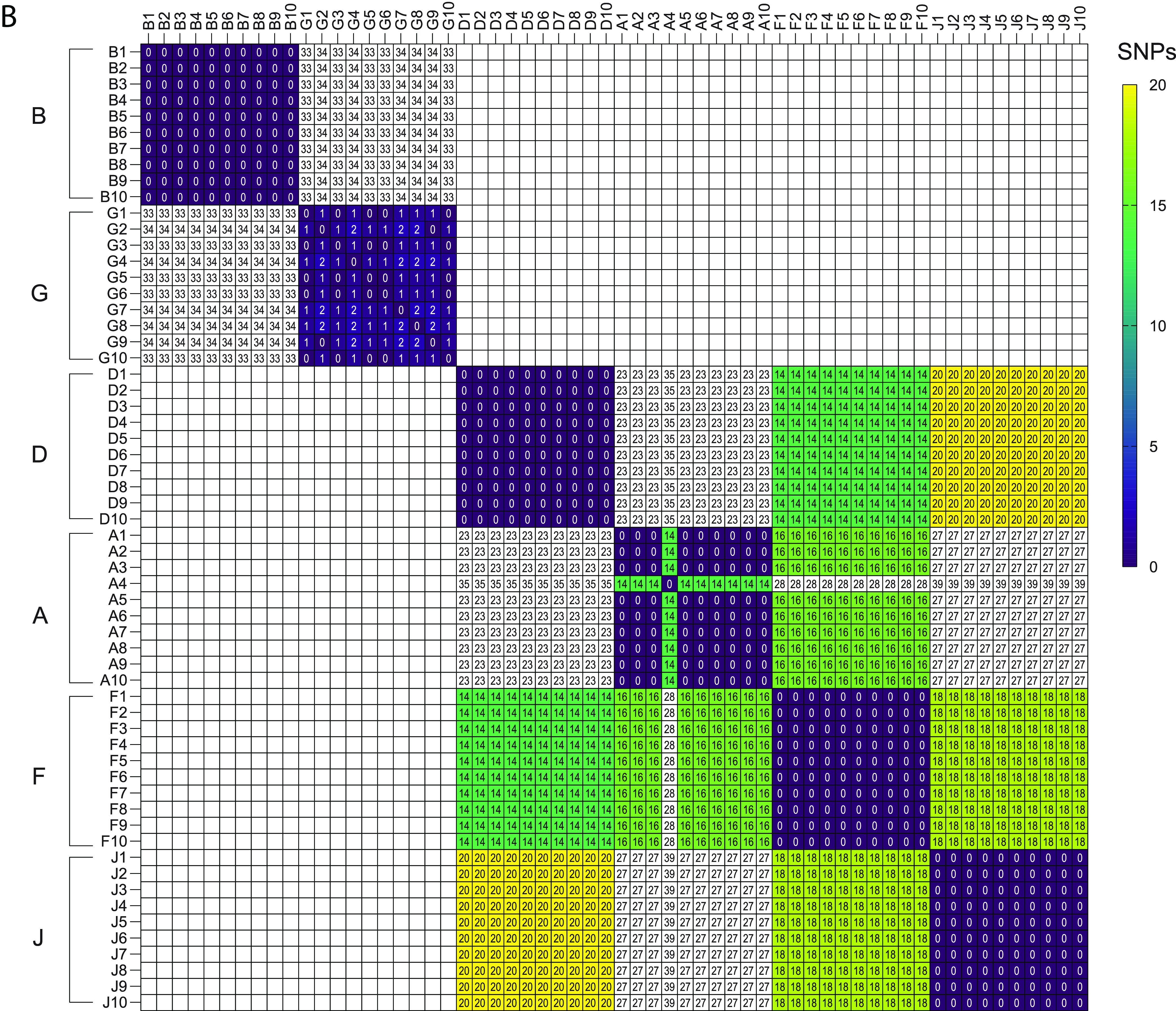
Comparisons of carbapenem-resistant K. pneumoniae strains by core genome SNP phylogeny and presence/absence of specific genes. (A) Summary of strain comparisons. Data were generated by analyses of short-read whole-genome sequences (Illumina HiSeq). The following comparisons between strains are presented (left to right): core genome SNP phylogenetic tree, carbapenemases, capsule (KL) types, and presence/absence of specific antibiotic resistance (red), virulence (green), and plasmid replication type (blue) genes. AMR determinants, virulence factors, and plasmid replicon genes were detected in well-resolved contigs with 100% coverage and >90% identity in short-read assemblies. The reference strain was K. pneumoniae 30660/NJST258_1 (GenBank assembly accession no. GCA_000598005.1). Taken together, these comparisons demonstrated within-host genetic diversity among strains from 5 of 6 patients (A, B, D, G, and J; no differences demonstrated for patient F). (B) Heat map of core genome SNPs. Strains from a given patient are bracketed and labeled with the patient designation. Numbers of core genome SNP differences are within matrix boxes. Strain A4 was the most distinct strain in within-patient comparisons, differing from other A strains by 14 core genome SNPs.

**(i) Evaluation of core genome SNP phylogeny and antibiotic resistance, capsular biosynthesis, virulence, and plasmid replicon genes.** We first performed core genome single nucleotide polymorphism (SNP) analysis. An alignment of core genome nucleotides was used to build a high-resolution SNP phylogenetic tree. Strains segregated into clade 1 (capsule type KL106; patients B and G) and clade 2 (capsule type KL107; patients A, D, F, and J) (Fig. 1A). Each patient’s strains clustered closely on the phylogenetic tree. We observed 100% bootstrap support in every cluster. Interstrain SNP differences are summarized in Fig. 1B. The most distinct strain in within-patient comparisons was A4, which differed from the other A strains by 14 core genome SNPs.

We next compared specific genome content of strains by surveying short-read WGS data for presence of genes involved in antibiotic resistance, capsular biosynthesis, and virulence and for genes associated with plasmid replicon types (Fig. 1A). All strains except G7 carried *bla*_KPC_. Within-host diversity in resistance, capsular or other virulence gene content was evident among strains from 5 patients (A, B, D, G, and J) (Fig. 1A); within-host diversity in presence or absence of capsular genes was evident in 2 patients (A and J; Table S2). Within-host diversity in plasmid replicon types was evident among strains from 2 patients (A and G) (Fig. 1A).

10.1128/mbio.02906-22.2 TABLE S2 Capsular gene mutations in carbapenem-resistant K. pneumoniae ST258-wzi154 (KL107) strains. ID, strain identification; cps, capsular polysaccharide; WT, wild-type; M, *wzc* mutation. *wzc* mutations are as follows: strain A4, proline-605 to glutamine; strains F1 to F10, tyrosine-710 frameshift; strains J4 and J8 phenylalanine-269 to leucine). +, presence of a specific capsular gene; −, absence of a specific capsular gene. Download Table S2, DOCX file, 0.03 MB. Copyright © 2022 Cheng et al. 2022 Cheng et al. https://creativecommons.org/licenses/by/4.0/ This content is distributed under the terms of the Creative Commons Attribution 4.0 International license.

**FIG 2:**
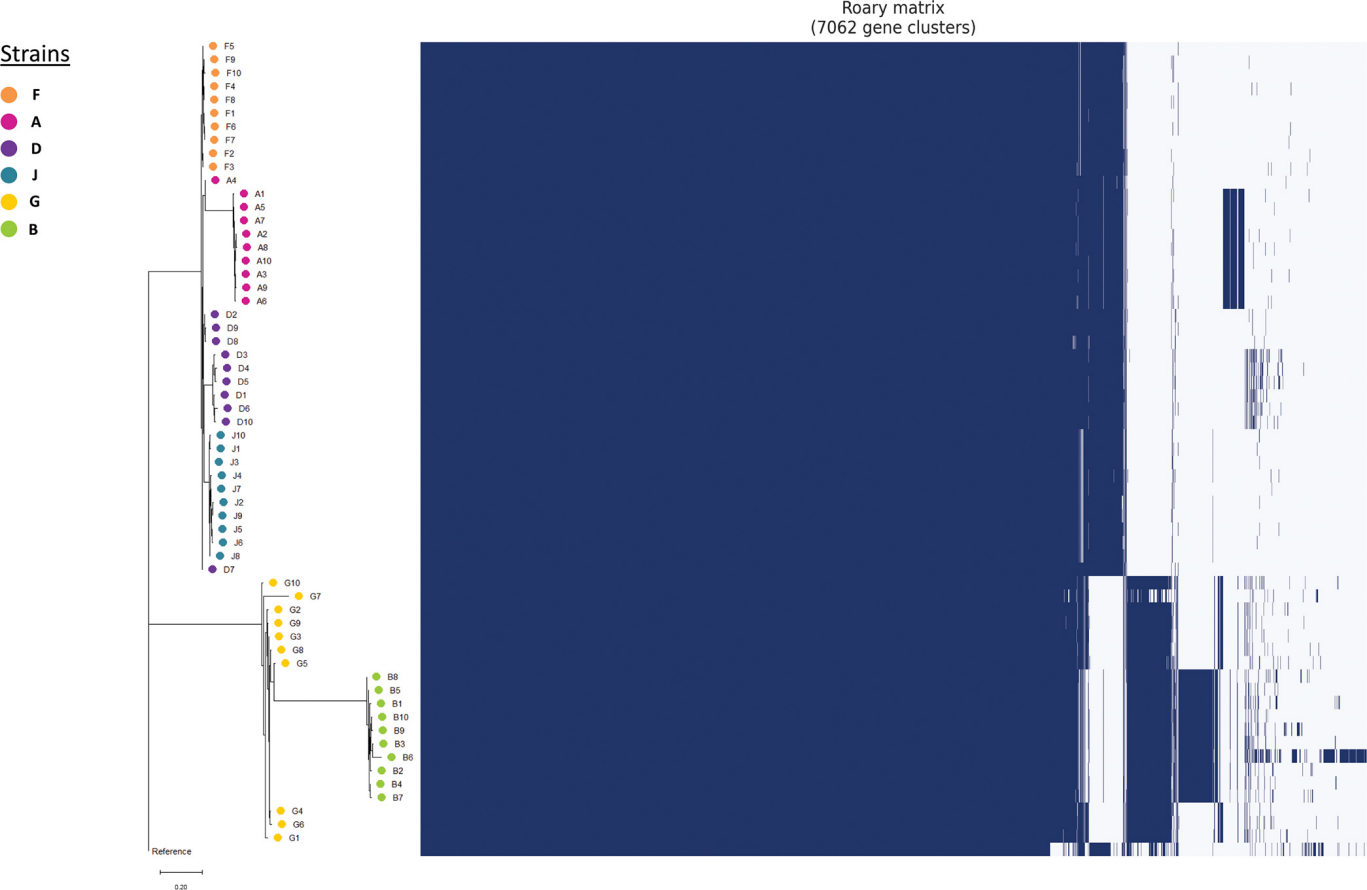
Pan-genome comparisons of carbapenem-resistant K. pneumoniae strains. Data were generated by analyses of short-read whole-genome sequences (Illumina HiSeq). The phylogenetic tree was constructed using data on presence/absence of 2,161 accessory genes (present in 1 to 57 genomes). The gene presence/absence matrix, covering 7,062 gene clusters, including 4,700 core genes (present in 60 or 61 genomes) and 201 soft-core genes (present in 57 to 59 genomes), is shown to the right of the phylogenetic tree. Blue, gene presence; white, gene absence. Within-host genetic diversity of each strain in all 6 patients was evident by pan-genome analyses. See Table S3 for sequence data for gene presence/absence matrices.

**(ii) Pan-genome analyses.** We constructed presence/absence matrices for 7,062 genes, including 4,700 core genes (present in ≥60 of 61 genomes), 201 soft-core genes (57 to 59 genomes), and 2,161 accessory genes (1 to 56 genomes) (Fig. 2; Table S3). Strains segregated by clade and by patient in the accessory gene phylogenetic tree. Within-host diversity was evident among strains from all patients.

10.1128/mbio.02906-22.3 TABLE S3 Pangenome matrices for carbapenem-resistant K. pneumoniae strains. Download Table S3, XLS file, 0.8 MB. Copyright © 2022 Cheng et al. 2022 Cheng et al. https://creativecommons.org/licenses/by/4.0/ This content is distributed under the terms of the Creative Commons Attribution 4.0 International license.

### Detailed descriptions of within-host CRKP genetic diversity in three patients.

We investigated strains from patients A, G, and J in greater detail. Strains from these patients were chosen since they demonstrated within-host differences in antibiotic resistance and virulence genes, the impact of which could be assessed in subsequent phenotypic assays. To generate complete chromosomal and plasmid reference sequences, we performed long-read sequencing (Oxford Nanopore WGS; MinION) on 2 strains from each of the 3 patients and constructed hybrid assemblies (strains A1, G1, and J1 [index strains] and A4, G7, and J2 [most distinct by short-read analyses]). Long-read WGS corroborated gene presence/absence data from short-read WGS and afforded accurate plasmid resolution (Table S4).

10.1128/mbio.02906-22.4 TABLE S4 Within-host, hybrid short- and long-read whole-genome sequence comparisons of carbapenem-resistant K. pneumoniae strains from three patients (A, G, and J). Data were generated using hybrid assemblies of long- and short-read WGSs. Plasmid information was mined using long-read WGS. Within-host differences in content are highlighted in bold. Chr, chromosome; KP1_RS17220, glycosyltransferase, LPS; KP1_RS17225, glycosyltransferase family 4 protein, LPS; KP1_RS17230, glycosyltransferase, LPS; KP1_RS17240, DUF4422 domain-containing protein, LPS; KP1_RS17355, phosphatase PAP2 family protein, capsule. Download Table S4, DOCX file, 0.02 MB. Copyright © 2022 Cheng et al. 2022 Cheng et al. https://creativecommons.org/licenses/by/4.0/ This content is distributed under the terms of the Creative Commons Attribution 4.0 International license.

**(i) Patient A.** Strain A4 was unique among A strains for lack of *tetA* (tetracycline resistance), virulence genes encoding aerobactin (*iucABCD*) and the salmochelin siderophore system (*iroBCDN*), and plasmid replicons IncFIB and IncFIC (Fig. 1A; Table S4). The loss of the plasmid associated with these replicons was confirmed by PCR. Long-read data verified that A4 lacked a 160-kb IncFI plasmid that carried *tetA* and several virulence genes. Strains A1 and A4 differed from one another by SNPs or deletions that resulted in nonsynonymous and synonymous changes in 16 and 9 genes, respectively; a SNP and deletion were also identified in intergenic regions (Table S5). Notable nonsynonymous mutations were observed in *wzc* (resulting in a glutamine-605-to-proline substitution), porin *ompK36* (C586→T; premature stop codon), ferric iron reductase *fhuF* (aspartic acid-10 to asparagine), and fimbrin adhesin *fimH* (glycine-96 to aspartic acid).

10.1128/mbio.02906-22.5 TABLE S5 Core genome single nucleotide polymorphisms and insertion-deletions in carbapenem-resistant K. pneumoniae strains from patients A (A1 and A4) and G (G1 and G4). Data were generated using hybrid assemblies of long- and short-read WGSs. K. pneumoniae 30660/NJST258_1 served as the reference genome. Note that no core genome variants were observed in strain J1 or J2. SNP, single nucleotide polymorphism; ins, insertion; del, deletion; Gln, glutamine; Leu, leucine; Gly, glycine; Asp, aspartic acid; Tyr, tyrosine; Arg, arginine; His, histidine; Pro, proline; Thr, threonine; Lys, lysine; Cys, cysteine; Val, valine; Trp, tryptophan; Asn, asparagine; Ile, isoleucine. Download Table S5, DOCX file, 0.03 MB. Copyright © 2022 Cheng et al. 2022 Cheng et al. https://creativecommons.org/licenses/by/4.0/ This content is distributed under the terms of the Creative Commons Attribution 4.0 International license.

**(ii) Patient G.** Strain G7 was unique among G strains for its lack of *bla*_KPC-2_ and *sul1* (sulfonamide) resistance genes and for the absence of a 167,851-bp IncF1K plasmid with IncFIB and IncFII replicons that carried *bla*_KPC-2_ (Fig. 1A). G7 differed from G1 by SNPs in 4 genes, 3 of which were nonsynonymous (Table S5); 5 other mutations were identified in intergenic regions. Strains G1 and G6 lacked *fimD* (encoding an adhesin) and the gene KP1_RS17225 (encoding a glycosyltransferase family 4 protein in the capsular polysaccharide [CPS] synthesis region), respectively (Fig. 1A). Strains G4 and G6 were notable for an IS*5* insertion in the promoter region of *ompK36* (Fig. S1), which has been shown to impact some broad-spectrum-beta-lactam and -carbapenem MICs (33).

10.1128/mbio.02906-22.8 FIG S1 *ompk36* sequences in carbapenem-resistant K. pneumoniae strains from patient G (G3, G4, and G6). Strains G4 and G6 carried mutations in the promoter region of *ompk36*. The *ompk36* sequence in strain G3 was similar to that of the remaining G strains. rbs, ribosome binding site. Download FIG S1, PDF file, 0.6 MB. Copyright © 2022 Cheng et al. 2022 Cheng et al. https://creativecommons.org/licenses/by/4.0/ This content is distributed under the terms of the Creative Commons Attribution 4.0 International license.

**FIG 3:**
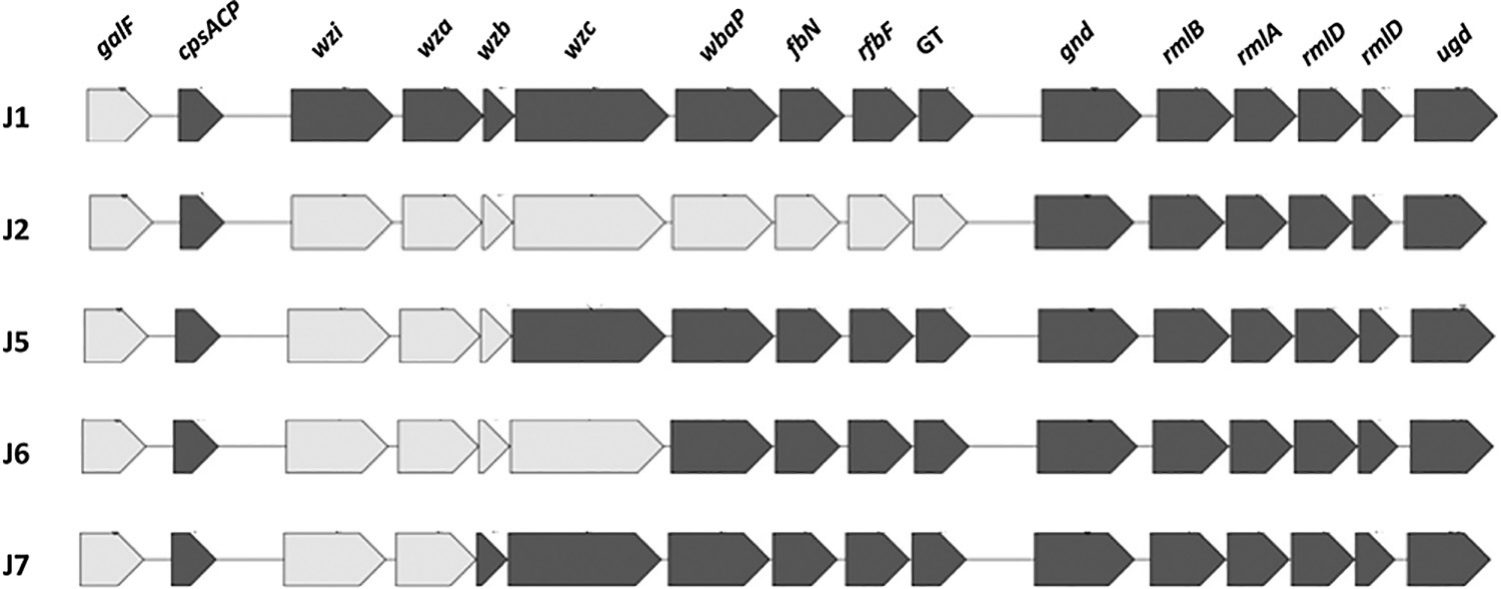
Capsular gene composition of carbapenem-resistant K. pneumoniae strains from patient J. Data are shown for representative J strains with given capsular gene composition. Based on gene deletions, J strains fell into 5 groups: (i) J1, J3, J4, J8, and J10; (ii) J2 and J9; (iii) J5; (iv) J6; and (v) J7. In addition to deletions shown in the figure, strains J4 and J8 also carried Phe262Leu substitutions in *wzc*. Therefore, J strains manifested 6 variations of the KL107 capsule. Gray and black shading indicates gene absence and presence, respectively. See Table 2 for further details on capsular gene mutations in strains from patients A, D, F, and J.

**(iii) Patient J.** J strains were most notable for 6 variations of KL107 capsule, associated with deletions and mutations of capsular biosynthesis genes (*galF*, *wzi*, *wbc*, *wzc*, and *wbaP*) (Table S2; Fig. 3). The predominant capsule had a 2.2-kb deletion encompassing *galF* (ΔKL107-2.2kb; J1, J3, and J10). Strains J4 and J8 had this 2.2-kb deletion, as well as a Phe269Leu substitution in *wzc*. Additional capsular gene deletions were detected in other J strains: ΔKL107-2.2kb+2.7kb (J7), ΔKL107-2.2kb+3.6 kb (J5), ΔKL107-2.2kb+6kb (J6), and ΔKL107-2.2kb+11kb (J2, J9). Strain J2 was unique among J strains for carrying a 4,097-bp sequence that did not include known antibiotic resistance or virulence genes. The best match with this sequence by BLAST nucleotide search was Escherichia coli OK15 plasmid unnamed4 (12,273 bp; GenBank no. CP081681.1; 100% query coverage; 83.62% identity). J4 was unique in lacking *bla*_TEM-150_, which encodes an extended-spectrum beta-lactamase.

### CRKP phenotypes.

There were no significant within-host differences in growth rates of any A, G, or J strains at 30 or 37°C in Mueller-Hinton (MH) medium or M9 minimal medium without or with a 100 μM concentration of the iron chelator deferoxamine.

**(i) Antibiotic susceptibility.** Antibiotic MICs against A, G, and J strains are presented in Table S6. Strain A4 was susceptible (MIC, 4 μg/mL) to tetracycline, whereas other A strains were resistant (MIC, 256 μg/mL), consistent with the absence and presence of *tetA*, respectively. Strain G7 was unique among G strains for susceptibility to meropenem (consistent with absence of *bla*_KPC-2_) and ceftazidime. Strains G4 and G6, which had an IS*5* insertion in the promoter region of *ompK36*, exhibited meropenem-vaborbactam MICs that were ≥4-fold higher than those exhibited by other G strains. *ompK36* expression by G4 and G6 was reduced ~60-fold and ~100-fold (*P = *0.004 for either strain, Mann-Whitney test), respectively, compared to other G strains by reverse transcription-PCR (RT-PCR) in both the presence and absence of meropenem-vaborbactam; OmpK36 production was significantly diminished in strains G4 and G6, as evident by sodium dodecyl sulfate–polyacrylamide gel electrophoresis (SDS-PAGE) (Fig. S2).

10.1128/mbio.02906-22.6 TABLE S6 Antibiotic MICs against carbapenem-resistant K. pneumoniae strains from three patients (A, G, and J). Susceptibility testing was performed using the Clinical and Laboratory Standards Institute reference broth microdilution method. MICs in bold in shaded boxes differ by ≥4-fold from MICs against other strains. MEM, meropenem; MVB, meropenem-vaborbactam; CAZ, ceftazidime; CZA, ceftazidime-avibactam; TET, tetracycline; GEN, gentamicin. Download Table S6, DOCX file, 0.02 MB. Copyright © 2022 Cheng et al. 2022 Cheng et al. https://creativecommons.org/licenses/by/4.0/ This content is distributed under the terms of the Creative Commons Attribution 4.0 International license.

10.1128/mbio.02906-22.9 FIG S2 *ompk36* expression and OmpK36 porin production by carbapenem-resistant K. pneumoniae strains from patient G (G1, G4, G6, and G7). Relative gene expression is shown on the left (as median fold change ± IQR) for each strain in the presence and absence of meropenem-vaborbactam (MV), as determined by RT-PCR. Protein production in absence of drug exposure is shown on the right, as determined by 12% sodium dodecyl sulfate–polyacrylamide gel electrophoresis. The “Ladder” lane contains size markers, in kilodaltons. Download FIG S2, PDF file, 0.04 MB. Copyright © 2022 Cheng et al. 2022 Cheng et al. https://creativecommons.org/licenses/by/4.0/ This content is distributed under the terms of the Creative Commons Attribution 4.0 International license.

**FIG 4:**
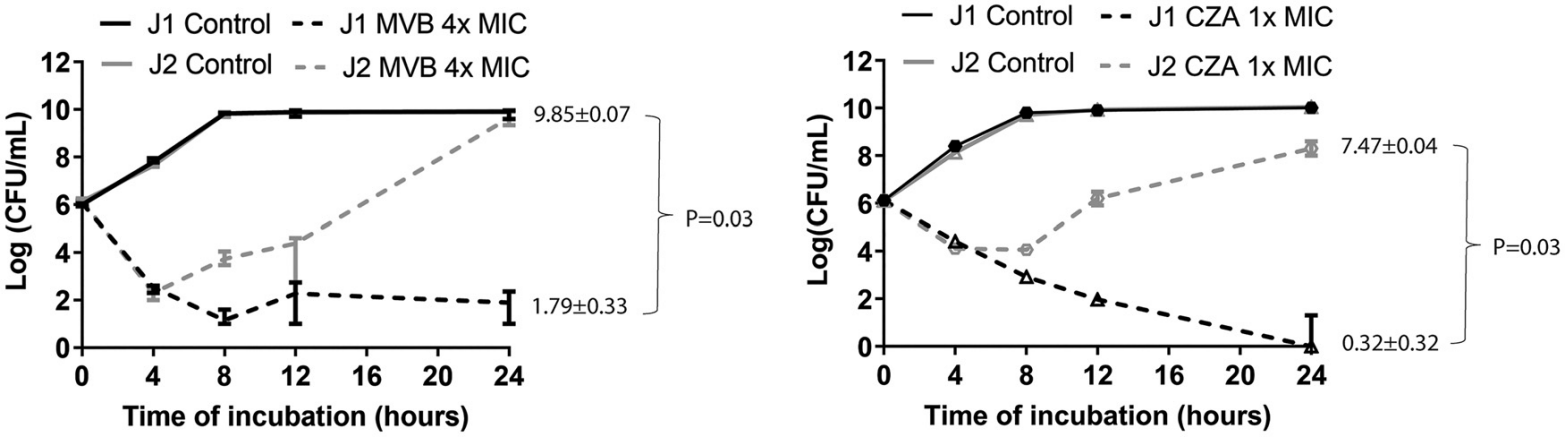
Meropenem-vaborbactam (MVB) and ceftazidime-avibactam (CZA) time-kill assays of carbapenem-resistant Klebsiella pneumoniae strains J1 and J2. MVB time-kill assays of strains J1 and J2 at 0× and 4× MIC are shown on the left. MVB MICs against both strains J1 and J2 were 0.06 μg/mL. Respective CZA MICs were 1 and 2 μg/mL. CZA time-kill assays at 0× and 1× MIC are shown on the right. Strains were incubated with 1× and 4× MIC of both drugs. For clarity in figures, data are not shown for MVB at 1× MIC or CZA at 4× MIC. MVB time-kill assays of J1 and J2 at 1× MIC did not differ significantly; both strains regrew after 4 h. CZA time-kill assays of the strains at 4× MIC did not significantly differ from those at 1× MIC. Data are medians and interquartile ranges from four independent experiments. *P* values were determined using the Mann-Whitney U test.

For J strains, there were no significant differences in MICs (Table S6). In a recent study, a capsule-deficient K. pneumoniae mutant strain had greater survival than a wild-type strain in bladder epithelial cells *in vitro* following exposure to meropenem-vaborbactam, in the absence of phenotypic resistance (34). We performed 24-h meropenem-vaborbactam and ceftazidime-avibactam time-kill experiments against strains J1 and J2, which were susceptible to the drugs according to the MICs. There was no difference between strains in initial killing at 4 h by either agent. However, between 4 and 24 h postexposure, J2 regrew in the presence of meropenem-vaborbactam and ceftazidime-avibactam; in contrast, there was no regrowth of J1 (35). At 24 h, J2 burdens were significantly greater than those of J1 for meropenem-vaborbactam at 4× MIC (Fig. 4). J2 isolates recovered after 24 h did not demonstrate increases in meropenem-vaborbactam or ceftazidime-avibactam MICs.

**(ii) CPS and mucoviscosity.** To investigate CPS content, we quantified uronic acid concentrations in representative strains from patients A (A1, A4, and A8), G (G1 and G7), and J (J1, J2, J5, J6, and J7) (Table 2). As expected, strains J2, J5, J6, and J7 had significantly lower CPS content than strain J1. We next evaluated mucoviscosity by measuring supernatant turbidity (optical density at 600 nm [OD_600_]) after low-speed centrifugation. Strains J2, J5, J6, and J7 exhibited significantly less mucoviscosity than strain J1. Differences in mucoviscosity were also clearly visible within tubes following centrifugation (Fig. S3). There were no significant within-host differences among A strains or G strains in either uronic acid concentrations or supernatant turbidity.

**TABLE 2:** Capsular genotypes and *in vitro* phenotypes of carbapenem-resistant K. pneumoniae strains from three patients^*a*^

Patient and strain	Capsule synthesis locus	CPS (uronic acid, nmol/mL)	Mucoviscosity (OD_600_)	% serum killing (mean ± SD)	% macrophage^*b*^ killing (mean ± SD)
A					
A1	KL107	142.1 ± 9.3	0.66 ± 0.05	52.8 ± 3.4	40.0 ± 4.3
A4	KL107	155.1 ± 20.3	0.72 ± 0.03	85.7 ± 6.5	35.6 ± 7.3
A8	KL107	142.8 ± 27.3	0.69 ± 0.08	86.9 ± 2.2	36.8 ± 4.8
*P* value		NS (0.07)	NS (0.20)	0.0001	NS (0.47)
G					
G1	KL107	91.8 ± 7.9	0.69 ± 0.03	56.8 ± 15.1	37.6 ± 4.9
G7	KL107	117.1 ± 15.0	0.65 ± 0.12	56.9 ± 14.0	22.7 ± 9.7
*P* value		NS (0.10)	NS (0.10)	NS (0.86)	NS (0.23)
J					
J1	KL107-2.2kb	75.6 ± 11.6	0.69 ± 0.01	85.0 ± 8.1	55.4 ± 8.6
J2	KL107-2.2-11kb	29.7 ± 5.6	0.41 ± 0.02	100 ± 0	52.8 ± 8.4
J5	KL107-2.2-3.6kb	38.0 ± 11.7	0.49 ± 0.02	100 ± 0	58.8 ± 9.0
J6	KL107-2.2-6kb	31.2 ± 7.1	0.44 ± 0.01	100 ± 0	56.3 ± 10.9
J7	KL107-2.2-2.7kb	35.9 ± 0.78	0.47 ± 0.01	100 ± 0	52.1 ± 9.2
*P* value		0.0001	<0.0001	0.01	NS (0.52)

a Data are presented as median ± interquartile range from at least 3 independent experiments. The differences in data between strains from the same patients were analyzed using the Kruskal-Wallis test (when >2 strains were tested) or the Mann-Whitney U tests (when 2 strains were tested). CPS, capsular polysaccharide; OD, optical density; SD, standard deviation; NS, nonsignificant.

b RAW264.7 macrophages.

10.1128/mbio.02906-22.10 FIG S3 Mucoviscosity of carbapenem-resistant K. pneumoniae strains from three patients (A, G, and J). Mucoviscosity was measured as OD_600_ of supernatants following low-speed centrifugation of strains in Luria-Bertani liquid medium. Photos of representative postcentrifugation tubes are shown. OD_600_ data (means ± standard errors of the means) for each strain appear below the photos. Strains with less mucoviscosity are less turbid in tubes and have lower OD_600_ than strains with higher mucoviscosity. Strain J2, J5, J6, and J7 exhibited significantly less mucoviscosity than J1 and strains from patients A and G. Download FIG S3, PDF file, 0.1 MB. Copyright © 2022 Cheng et al. 2022 Cheng et al. https://creativecommons.org/licenses/by/4.0/ This content is distributed under the terms of the Creative Commons Attribution 4.0 International license.

**(iii) Serum and macrophage killing.** CPS has been shown to protect bacteria from complement-mediated killing in serum (36). Strains J2, J5, J6, and J7 were completely killed upon serum incubation; these strains were significantly more susceptible to serum killing than strain J1 or strains from patients A (A1, A4, and A8) or G (G1 and G7) (Table 2). Strains A4 and A8 were significantly more susceptible to serum killing than strain A1. There were no significant differences in susceptibility to serum killing among G strains. There were no significant within-host strain differences in susceptibility to macrophage killing.

**FIG 5:**
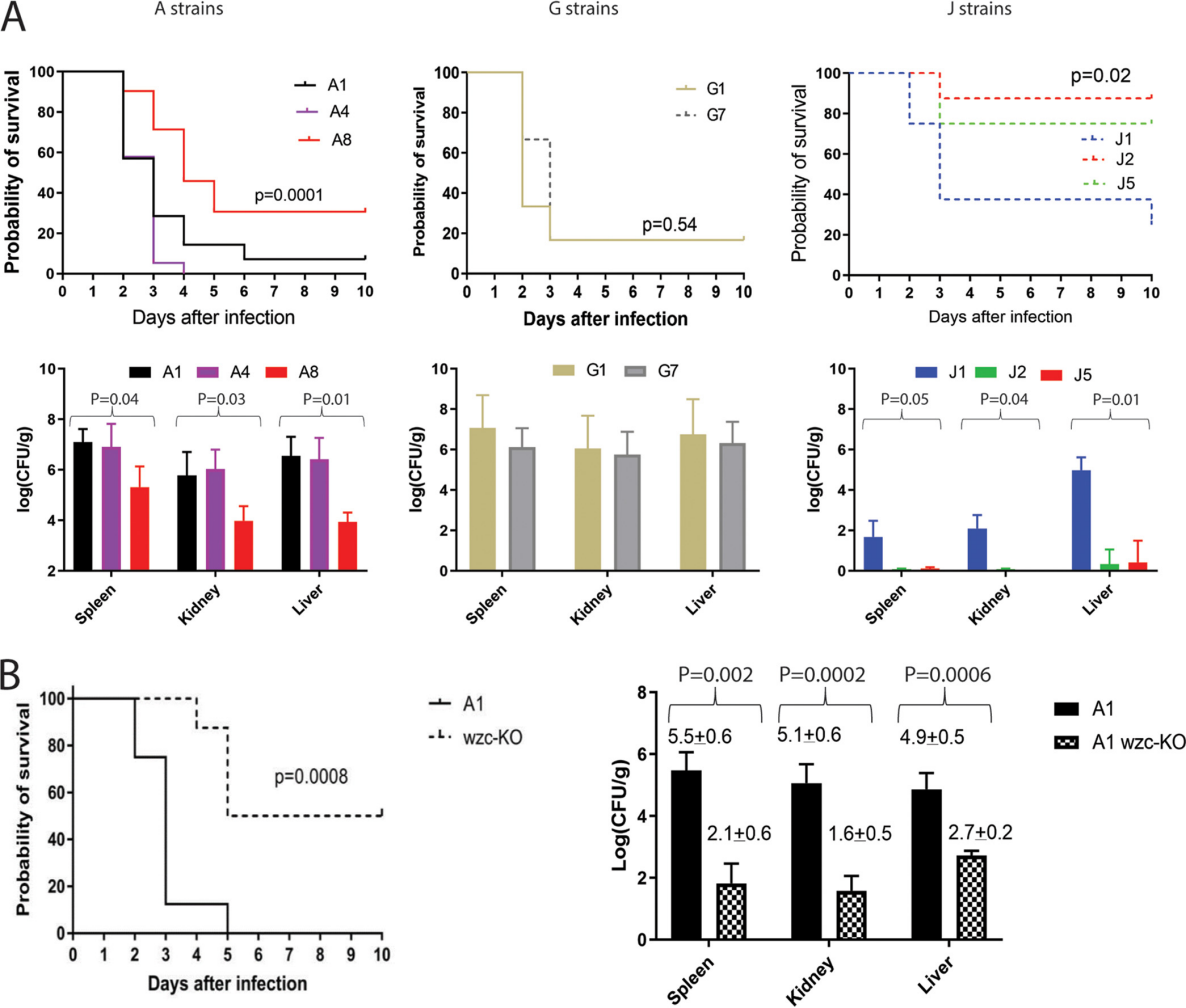
Mortality and tissue burdens of immunosuppressed mice during hematogenously disseminated K. pneumoniae infections. (A) Mortality and tissue burden data for mice infected with strains from patients A, G, and J. (B) Mortality and tissue burden data for mice infected with a parent strain (black bars) or *wzc* isogenic null mutant strain (checkerboard bars). In the latter experiments, the parent strain was pCasKp-harboring A1, and the *wzc*-null mutant strain was created in the parent background by a CRISPR-Cas9 method. As shown, there were significant differences in outcomes (mortality, tissue burdens) among mice infected with strains from patients A and J, and with the *wzc*-null mutant. There was no difference in outcomes among mice infected with strains from patient G. Cyclophosphamide- and cortisone-treated mice were infected intravenously with the indicated strains (mortality studies, 1 × 10^5^ CFU per strain; tissue burden studies, 1 × 10^4^ CFU per strain). Tissue burdens (presented as means and standard errors of the means) were determined in spleens, kidneys, and livers at 48 h. Differences in mortality were compared using the Mantel-Cox log rank test. Differences in tissue burdens were compared using the Kruskal-Wallis test (for A and J strains; comparisons between 3 strains) or Mann-Whitney U test (for G strains and parent/*wzc*-null mutant strains; comparisons between two strains).

**(iv) Virulence during bloodstream infections of mice.** To examine virulence *in vivo*, we infected cyclophosphamide- and cortisone-treated mice intravenously with strains from patients A (A1, A4, and A8), G (G1 and G7), or J (J1, J2, and J5). Outcomes, measured as mortality or tissue burdens in spleens, kidneys, and livers, were worse for mice infected with strains A1 and A4 than A8 (Fig. 5A). There were no differences in outcomes of mice infected with G1 or G7. Mice infected with strain J1 had worse outcomes than mice infected with J2 or J5, by both mortality and tissue burden endpoints.

To confirm that deletion of *wzc*, as observed in J2 and several other J strains, contributed to attenuated virulence, we used a CRISPR-Cas9 method to create an isogenic gene disruption strain in the background of A1 and infected immunosuppressed mice intravenously. We used strain A1 rather than J1 to create the mutant because the latter contains other capsular mutations that might confound results. The *wzc* mutant and paired A1 parent strains exhibited similar growth rates *in vitro*. The null mutant had significantly reduced CPS content, and it caused significantly attenuated mortality and lower burdens within spleens, kidneys and livers of mice during BSIs than did the parent strain (Fig. 5B).

### WGSs of CRKP from seeded blood cultures.

To assess if CRKP genetic diversity might arise during growth *in vitro*, we seeded sterile blood culture bottles and MH broth with index strains A1, G1, and J1. We performed short-read sequencing (Illumina HiSeq) on 10 strains recovered from randomly chosen colonies from each index strain following incubation in blood cultures at 37°C for a median of 3 days. Likewise, we performed short-read sequencing on two strains randomly chosen from index strains following incubation in MH broth for 24 h at 37°C. Strains from blood cultures and MH broth were indistinguishable from the respective index strains with regard to growth rates *in vitro* and antibiotic MICs, as well as core genome SNPs, gene and plasmid content, and pangenome analyses (Table S7).

10.1128/mbio.02906-22.7 TABLE S7 Whole-genome sequence comparisons of carbapenem-resistant K. pneumoniae strains from seeded blood culture bottles. We isolated strains from 10 colonies grown from index strains A1, G1, and J1 in blood culture bottles, as well as from 2 colonies from index strains grown in Mueller-Hinton (MH) broth. Strains are labeled as follows, using index strain A as an example: A1, index strain; A1_1, the first strain chosen following incubation in blood culture bottles; A1_2, the second strain chosen following incubation in blood culture bottles, etc.; AMH1, the first strain chosen following incubation in MH broth; AMH2, the second strain chosen following incubation in MH broth. Tabs in the Excel file are as follows. (i) Patient A SNP. Core genome SNP matrix for A strains. (ii) Patient G SNP. Core genome SNP matrix for G strains. (iii) Patient J SNP. Core genome SNP matrix for J strains. (iv) Patient J capsule. Data for capsular genes for J strains. (v) Control resistome. Data for presence/absence of resistance genes for A, G, and J strains. Strains from blood cultures and MH broth were indistinguishable from respective index strains with regard to growth rates *in vitro* and antibiotic MICs, as well as core genome SNPs, gene and plasmid content, and pangenome analyses. Download Table S7, XLSX file, 0.02 MB. Copyright © 2022 Cheng et al. 2022 Cheng et al. https://creativecommons.org/licenses/by/4.0/ This content is distributed under the terms of the Creative Commons Attribution 4.0 International license.

## DISCUSSION

This study is the first genome-wide analysis of within-host diversity of K. pneumoniae recovered from individual patients with BSIs. We showed that positive blood cultures comprised genetically heterogeneous ST258 K. pneumoniae populations, with strains differing with regard to gene mutations, presence or absence of specific genes (including those involved in antibiotic resistance, capsular synthesis, and other processes relevant to pathogenesis), and plasmid or pan-genome gene content. Moreover, we demonstrated that genetically diverse strains exhibited unique phenotypes that are potentially important during BSIs, including differences in antibiotic responses, CPS and mucoviscosity, resistance to serum killing, and ability to cause organ infections or mortality *in vivo*. This diversity was not appreciated by standard clinical microbiology laboratory approaches, in which a single strain was selected from positive blood cultures for further characterization. Rather, diversity was unmasked only by studying strains from multiple, morphologically indistinguishable colonies. Our data suggest a new, population-based paradigm for CRKP BSIs.

To our knowledge, only one other study has assessed bacterial genetic diversity during BSIs by whole-genome sequencing of strains from morphologically indistinguishable colonies. In that study, genetic variants of the same ST were identified in 36% of patients with Staphylococcus aureus bacteremia (12). The investigators did not report whether genetic variant strains exhibited distinct phenotypes. The higher prevalence of genetic diversity we observed may reflect the sequencing of strains from 10 rather than 3 to 5 colonies, differences in comparative genomic analytic methods, and/or particularly strong selection pressures encountered by patients with CRKP infections. Indeed, patients in our study had significant medical conditions and surgical interventions, as well as recurrent hospitalizations. Each of them was heavily antibiotic experienced, and five of six patients had had previous invasive infections or colonization with CRKP. Pressures encountered by the patients may have selected for outgrowth of variant strains within the population that were better able to persist and proliferate as opportunistic pathogens (37, 38).

Genetic variants are increasingly recognized among bacterial strains during colonization and chronic infections of nonsterile sites, including the GI tract (Helicobacter pylori, *Enterococcus* spp.) (16, 17, 19, 37), lungs (Pseudomonas aeruginosa, *Burkholderia* spp., Mycobacterium tuberculosis) (14, 18, 20, 39), nasal cavity (S. aureus) (12, 13, 22), and skin (Staphylococcus epidermidis) (40). Emergence of diversity might be expected during such long-term interactions with the host (19, 21, 37, 38), but it is more surprising in acute infections of a putatively sterile site. In one study, capsular gene mutant subpopulations were identified in 10% of K. pneumoniae-positive urine cultures, including samples associated with acute urinary tract infections (UTIs) in 2 patients (34). In fact, diversity may have been underestimated in these cultures, since investigators screened for hypermucoid phenotypes, rather than employing a sequence-first approach.

The capsule is the major virulence factor in K. pneumoniae and other *Enterobacterales* (36). Capsular gene mutations or deletions were identified in strains from 4 of 6 patients (A, D, F, and J), each of which had a KL107 capsule type. In 3 patients, there was within-host diversity of capsular mutant strains, including variants with nonsynonymous *wzc* SNPs (A and J) and various gene deletions (D and J). J strains with extensive capsular gene deletions were significantly attenuated in CPS content and mucoviscosity, more susceptible to serum killing, and less virulent during hematogenously disseminated infections of mice relative to strain J1, which had more limited capsular deletion. Using isogenic ST258 strains, we showed that disruption of *wzc*, as in several J strains, led to significantly reduced CPS content and attenuated virulence during mouse BSIs. Capsular gene mutant K. pneumoniae strains belonging to various STs that exhibit hypermucoid or hypomucoid phenotypes have emerged repeatedly and independently in clinical cultures; 10% of ST258, clade 2 K. pneumoniae genomes in the NCBI RefSeq database carry nonsynonymous mutations in *wzc* or *whaP* capsule genes (34). Our findings of attenuated virulence for hypomucoid strains with *wzc* disruption or more extensive capsular mutations are broadly consistent with prior observations that hypermucoid K. pneumoniae caused greater lethality in mouse BSIs (34).

We showed that strain J2, which had the largest capsular gene deletion among J strains, regrew after 4 to 24 h of exposure to meropenem-vaborbactam or ceftazidime-avibactam *in vitro*, despite MICs in the susceptible range. In contrast, the drugs exerted prolonged bactericidal activity against strain J1. These findings are consistent with those from a previous study in which a capsule-deficient K. pneumoniae strain was more tolerant than a wild-type strain to meropenem-vaborbactam in bladder epithelial cells *in vitro* (34). It is plausible that capsular mutations that reduce intrinsic K. pneumoniae virulence during BSIs may afford advantages during antibiotic treatment. The data carry potential clinical importance, since meropenem-vaborbactam or ceftazidime-avibactam are drugs of choice against KPC-producing CRKP infections (41). Of note, the meropenem-vaborbactam-tolerant, capsule-deficient K. pneumoniae strain from the earlier study demonstrated enhanced virulence in untreated mice with chronic UTIs (34). Therefore, capsular mutations that reduce fitness in some environments *in vivo* may be advantageous in other environments, independent of contributions to antibiotic responses. Taken together, data in the present and past studies attest to the complex, multifactorial nature of K. pneumoniae virulence. Along these lines, strain A4 lacked numerous virulence genes found in other A strains, including those encoding aerobactin and siderophores, but it nevertheless caused higher tissue burdens and greater mortality during mouse BSIs.

Our results challenge the single-organism hypothesis of bacteremia, in which infections are typically ascribed to one strain that accesses the bloodstream through a bottleneck (38). It is unclear whether within-host diversity here stemmed from one-time inoculation of a mixed population from the GI tract or other portal, serial introduction of different strains, or evolution within the bloodstream. For several reasons, we believe that diversity was most likely generated within the GI tract or other site of colonization or persistent infection. First, in control experiments, incubation of index strains A1, G1, and J1 in sterile blood culture bottles did not lead to genetic or phenotypic changes among recovered strains. Second, genetic and phenotypic diversity is well described in GI-colonizing populations of enteric bacteria (17, 19, 37, 38). Most CRKP BSIs are caused by GI-colonizing strains, and, as in our experience, patients at risk for CRE infections typically encounter intense and long-term selection pressures for microbial diversification, including repeated exposure to broad-spectrum antibiotics (6–8, 42). Finally, our detection of mutations in biologically plausible targets that were previously described among K. pneumoniae clinical isolates recovered from diverse body sites, such as antibiotic resistance, capsular biosynthesis, and porin genes, supports the validity of our findings and suggests that they reflect diversity *in vivo* (6, 33, 34, 42). In the future, metagenomic sequencing may afford in-depth coverage of microbial variants in samples directly from sites of infection. Currently, however, metagenomic sequencing of bacteria within blood is limited by overwhelming predominance of host DNA and low concentrations of microbial DNA, need for target amplification, and challenges in assigning sequence variations to individual strains (38, 43). Follow-up studies are warranted to compare CRKP diversity at GI and other sites of colonization with that observed during BSIs and to investigate BSIs caused by other K. pneumoniae STs and other bacterial species.

The clinical significance of CRKP diversity shown here is unknown and also merits further investigation. A strength of our study design is that CRKP strains were collected as blood cultures were being processed according to standard clinical microbiology laboratory practices. The possible impact of unrecognized diversity is highlighted in patient G, who would have been diagnosed with BSI due to carbapenem-susceptible K. pneumoniae rather than CRKP if G7 had been randomly selected as the index strain instead of G1. Indeed, studies of patients diagnosed with more susceptible K. pneumoniae BSIs than were identified in our patients would allow investigators to address how often clinical laboratories fail to identify resistant variants (i.e., heteroresistance) within the population and whether such events lead to treatment failures (44). Future longitudinal studies should assess treatment responses, patient outcomes, and endpoints such as emergence of *de novo* antibiotic resistance, selection for preexisting resistant strains, and adaptive bacterial evolution. If bacterial diversity is proven to be clinically relevant, microbiology laboratory practices will need to be modified. At present, our findings and those of studies showing bacterial genetic diversity at nonbloodstream sites have important implications for molecular epidemiologic investigations of infectious outbreaks and nosocomial transmission of pathogens. Data here and in our previous study of clinical and environmental *Mucorales* suggest caution in relying upon core genome SNP phylogeny as the sole tool in defining differences between strains (45). In both studies, comprehensive pan-genome analyses revealed variations that were not apparent with core genome SNP comparisons.

In conclusion, we identified genotypic and phenotypic variant strains of ST258 K. pneumoniae from blood cultures of individual patients. Clinical implications of such genetic and phenotypic diversity during BSIs and other infections will be defined in the years to come, with potentially profound implications for medical, clinical microbiology laboratory and infection prevention practices, and for better understanding of emergence of antibiotic resistance and pathogenesis.

## MATERIALS AND METHODS

### Strains and growth conditions.

Six patients with CRKP BSIs were identified at the University of Pittsburgh Medical Center between April 2017 and August 2018. First positive blood culture bottles from each patient during the study period were obtained from the clinical laboratory immediately after routine microbiological workup. We streaked 25 μL of broth from culture bottles onto blood agar plates (5% sheep blood in tryptic soy agar), and randomly picked 9 morphologically indistinguishable single colonies. Each colony was subcultured onto MH agar plates. Following overnight growth at 37°C, a single strain per original colony underwent DNA extraction (PureLink genomic DNA minikit; Fisher); remaining colonies and confluent growth were frozen in 20% glycerol at −80°C. We also obtained the index strain from each patient that was isolated by the clinical laboratory, and we extracted DNA and made frozen stock using the methods above. “Strain” is this study is defined as a CRKP isolate from a single colony that underwent WGS analyses.

### Short-read WGS and analyses.

Sixty strains (10 per patient) were sequenced using Illumina HiSeq. Raw short-reads were quality trimmed and *de novo* assembled into contigs using Shovill v1.1.0 (https://github.com/tseemann/shovill). We evaluated genome assembly quality by Quast v5.0.2 (46). Draft genomes were screened for contamination and annotated using MASH v2.3 and Prokka v1.14.5, respectively (47, 48). Species identification, K and O typing, and sequence typing were performed using Kleborate v2.0.4 (49). Antibiotic resistance, virulence, and plasmid replicon type genes were detected in the assembled genomes by ABRicate v1.1.0, using the NCBI database, the Virulence Factor Database (VFDB; http://www.mgc.ac.cn/VFs/), and the PlasmidFinder database (https://cge.food.dtu.dk/services/PlasmidFinder/). In addition, read-based antimicrobial resistance (AMR) detection was performed using Resfinder4.0. Core genome SNP analysis was undertaken by Snippy v4.6.0 (https://github.com/tseemann/snippy), using K. pneumoniae 30660/NJST258_1 (GenBank assembly accession no. GCA_000598005.1) as the reference. Recombination detection/masking was performed prior to phylogenetic analyses using Gubbins v.3.2.1 (50). The resulting core SNPs were concatenated and used to infer a phylogenomic tree by using RAxML v8.0 under the general time-reversible model with 1,000-bootstrap replicates (51). The phylogenetic tree and associated metadata were visualized using iTOL v5.6.3 (52). A pan-genome was constructed by Roary v3.13.0 using default settings, paralog splitting on, and 95% minimum identity for BLASTp (53). Core genes were defined as those present in 99% of strains, which prevented the outgroup strain from affecting core genome estimation. Plasmid content in strains from patient A was verified by PCR using the primers Tet(A)F (TGTCCACCAACTTATCAGTGA) and Tet(A)R (TGCCCCTGACGTTCCTCAT).

### Long-read WGS and hybrid assemblies.

DNA was isolated from overnight cultures using a MasterPure Gram-positive DNA purification kit (Epicentre, USA). Nanopore libraries were prepared using a ligation sequencing kit (SQK-RBK109) and sequenced by an R9.4 flow cell using a MinION MK1B device. Hybrid assemblies were produced by Unicycler v0.4.8 using default parameters (54) and visualized using Bandage v0.9.0 (55). Variant calling was performed by Snippy v4.6.0 (https://github.com/tseemann/snippy) with default settings.

### Antibiotic susceptibility testing and time-kill assays.

MICs were determined by the Clinical and Laboratory Standards Institute reference broth microdilution method. K. pneumoniae ATCC 700603 served as an internal quality control. Time-kill assays (4 replicates) were performed as previously described, using a single bacterial colony grown overnight in 4 mL MH broth (56).

### *ompK36* RT-PCR and SDS-PAGE.

RT-PCR and SDS-PAGE were performed as previously described (56). Relative quantities of mRNA from each gene were determined by comparative cycle threshold (*C_T_*). Expression of *ompK36* was normalized to that of *recA* and *rpoD*. Outer membrane proteins were analyzed in 12% SDS-PAGE and stained with a silver stain kit (Bio-Rad).

### CPS quantitation.

CPS was extracted and uronic acid concentrations were quantified (nanomoles per 10^9^ CFU) using established methods (57).

### Mucoviscosity.

Mucoviscosity was assessed by low-speed centrifugation of CRKP strains grown overnight in Luria-Bertani liquid medium at 37°C. Cultures were normalized to an OD_600_ of 1 and centrifuged at 100 × *g* for 20 min at 22°C (Marathon 3000R, swinging bucket; Fisher Scientific). The OD_600_ of the supernatant was determined (BioMate 3 Thermo Spectronic; Fisher Scientific).

### Serum killing (58).

Overnight cultures were diluted 1:100 and grown to mid-exponential phase. An inoculum of 2.5 × 10^4^ CFU of bacteria in 25 μL was mixed with 75 μL human serum from healthy volunteers (catalog no. BP2657100; Fisher Scientific). The mixture was incubated at 37°C, and aliquots were taken at baseline and 1 h to calculate viable CFU. Average percent survival was plotted against time.

### Macrophage killing (59).

*In vitro* killing of strains was investigated using the RAW264.7 macrophage cell line. In a 96-well plate, 8 × 10^4^ macrophages were resuspended in Dulbecco’s modified Eagle medium (DMEM), seeded in 3 wells, and incubated overnight at 37°C. After 12 h, 1.6 × 10^6^ CRKP CFU were added onto the monolayer at time zero and incubated at 37°C. Bacteria were washed 3 times with DMEM, and macrophages were lysed with H_2_O. The number of intracellular bacteria was determined by serial dilutions. Intracellular killing was based on the decrease in the number of viable bacteria 30 min after initial coincubation.

### Mouse infections.

Male ICR CD1 mice weighing 20 to 25 g (Harlan) were immunosuppressed with 2 doses of intraperitoneal cyclophosphamide (150 mg/kg of body weight 4 days prior to infection and 100 mg/kg 1 day prior to infection) and 2 doses of subcutaneous cortisone (20 mg/kg 4 days and 1 day prior to infection). For mortality studies, mice (8 to 12/group) were infected via lateral tail vein with 1 × 10^5^ CRKP suspended in 100 μL of saline. Mice were followed until they were moribund, at which point they were sacrificed, or for 10 days. For tissue burdens, 1 × 10^4^ CRKP were injected via the lateral tail vein. Mice (8 to 12/group) were sacrificed at 48 h postinfection.

### CRISPR-Cas9 deletion of *wzc*.

CRISPR-Cas9-mediated *wzc* deletion was conducted as previously described (60). *wzc*-specific guide RNA (gRNA) (GGTTTTGATGTAATACAGAG) was designed and inserted into plasmid vector pSgKp-Rif (61). *wzc* gRNA pSgKp-Rif and a 90-nucleotide (nt) synthesized template (AAAGCATGGGCGGAAAAATTAGCAAGTTAATTCAGGAAAATATACAGAAGTGTTTTCAACAAAAGCCGGATTTAGATAAATATTTAGATT) were electroporated into pCasKp-harboring CRKP A1 competent cells to create the knockout strain.

### WGSs of CRKP from seeded blood cultures.

We inoculated 2.5 to 5 CFU of index strains A1, G1, and J1 into a blood culture bottle containing 5 mL of sterile blood from a healthy volunteer and into 5 mL of MH broth. Blood culture bottles and MH broth were incubated with shaking (250 rpm) at 37°C until Gram-negative bacteria were evident by Gram staining (medians, 3 days and 24 h, respectively), after which aliquots were streaked on blood agar plates. For each patient, resultant colonies were morphologically similar. Single strains were isolated from 10 randomly selected blood culture colonies and two randomly selected MH broth colonies for Illumina HiSeq sequencing, as described above, and for measurements of growth rates *in vitro* and antibiotic MICs.

### Statistical analysis.

Experiments that produced quantitative data were performed at least in triplicate. Data are presented as means and standard error for symmetric data and as medians and interquartile ranges (IQR) for asymmetric data. Bacterial tissue burdens in mice were log-transformed prior to data analysis. Statistical analyses were performed using GraphPad Prism version 9.4. Mann-Whitney U and Kruskal-Wallis tests were used for statistical comparisons of 2 and >2 groups, respectively. Survival curves were calculated according to the Kaplan-Meier method using Prism and compared using Newman-Keuls analysis. For all analyses, a *P* value of <0.05 was considered significant.

### Data availability.

Raw reads for 60 ST258 K. pneumoniae strains from 6 patients and 30 strains from control blood culture seeding experiments were submitted to the NCBI Sequence Read Archive (BioProject numbers PRJNA826066 and PRJNA884340, respectively).
