# Supplementary material for: Within-Host Genotypic and Phenotypic Diversity of Contemporaneous Carbapenem-Resistant Klebsiella pneumoniae from Blood Cultures of Patients with Bacteremia
Source: mBio. 2022 Nov 29;13(6):e02906-22. doi: 10.1128/mbio.02906-22 (PMC9765435; doi:10.1128/mbio.02906-22)
Supplement: TABLE S1 [file mbio.02906-22-s0004.docx]

**Supplemental Table 1. Assembly data for carbapenem-resistant *Klebsiella pneumoniae* strains.**

| **Patient** | **Assembly ID** | **Number of contigs** | **N50** | **GC (%)** | **Total length (bp)** |
| --- | --- | --- | --- | --- | --- |
| **A** | A1 | 95 | 204929 | 57.07 | 5741056 |
|  | A2 | 108 | 203896 | 57.08 | 5754913 |
|  | A3 | 102 | 184789 | 57.08 | 5742016 |
|  | A4 | 80 | 210218 | 57.28 | 5586094 |
|  | A5 | 94 | 210218 | 57.08 | 5741860 |
|  | A6 | 96 | 191749 | 57.08 | 5741718 |
|  | A7 | 95 | 204929 | 57.07 | 5740761 |
|  | A8 | 96 | 204929 | 57.08 | 5743308 |
|  | A9 | 92 | 210218 | 57.08 | 5740925 |
|  | A10 | 95 | 204929 | 57.08 | 5741652 |
| **B** | B1 | 145 | 198995 | 56.58 | 5989234 |
|  | B2 | 125 | 179640 | 56.64 | 5980608 |
|  | B3 | 154 | 179282 | 56.62 | 5998834 |
|  | B4 | 143 | 179640 | 56.6 | 5985189 |
|  | B5 | 139 | 180217 | 56.62 | 5975643 |
|  | B6 | 627 | 136604 | 56.14 | 6445292 |
|  | B7 | 140 | 179640 | 56.59 | 5987093 |
|  | B8 | 148 | 179640 | 56.6 | 5981479 |
|  | B9 | 198 | 179516 | 56.51 | 6042780 |
|  | B10 | 137 | 179640 | 56.63 | 5972124 |
| **D** | D1 | 153 | 210218 | 57.2 | 5644669 |
|  | D2 | 83 | 224985 | 57.28 | 5594731 |
|  | D3 | 170 | 224985 | 57.18 | 5662925 |
|  | D4 | 146 | 224985 | 57.21 | 5641352 |
|  | D5 | 171 | 210218 | 57.18 | 5663130 |
|  | D6 | 142 | 210218 | 57.2 | 5635671 |
|  | D7 | 85 | 204928 | 57.28 | 5588732 |
|  | D8 | 88 | 209734 | 57.28 | 5591408 |
|  | D9 | 87 | 210218 | 57.28 | 5593503 |
|  | D10 | 143 | 224988 | 57.21 | 5634115 |
| **F** | F1 | 89 | 154552 | 57.27 | 5581628 |
|  | F2 | 97 | 204928 | 57.28 | 5591963 |
|  | F3 | 84 | 163077 | 57.25 | 5574418 |
|  | F4 | 96 | 204928 | 57.28 | 5590330 |
|  | F5 | 88 | 141542 | 57.26 | 5582580 |
|  | F6 | 91 | 204928 | 57.28 | 5587202 |
|  | F7 | 87 | 210218 | 57.28 | 5583857 |
|  | F8 | 81 | 203896 | 57.29 | 5576317 |
|  | F9 | 79 | 220597 | 57.3 | 5577027 |
|  | F10 | 87 | 204928 | 57.3 | 5577662 |
| **G** | G1 | 181 | 148802 | 57.04 | 5774161 |
|  | G5 | 159 | 155870 | 57.04 | 5771492 |
|  | G7 | 128 | 171115 | 57.09 | 5651044 |
|  | G2 | 162 | 146872 | 57.06 | 5761469 |
|  | G8 | 157 | 148802 | 57.07 | 5760327 |
|  | G9 | 140 | 146872 | 57.08 | 5736849 |
|  | G3 | 137 | 167839 | 57.09 | 5740321 |
|  | G4 | 139 | 148802 | 57.1 | 5740626 |
|  | G10 | 163 | 155373 | 57.08 | 5740252 |
|  | G6 | 146 | 146383 | 57.06 | 5751052 |
| **J** | J1 | 97 | 152548 | 57.3 | 5568939 |
|  | J2 | 90 | 204581 | 57.32 | 5559467 |
|  | J3 | 86 | 204936 | 57.27 | 5569594 |
|  | J4 | 83 | 204581 | 57.28 | 5561739 |
|  | J5 | 89 | 191193 | 57.27 | 5564664 |
|  | J6 | 92 | 204936 | 57.32 | 5562457 |
|  | J7 | 92 | 204581 | 57.31 | 5566422 |
|  | J8 | 99 | 141661 | 57.3 | 5569269 |
|  | J9 | 89 | 204936 | 57.34 | 5558070 |
|  | J10 | 92 | 204936 | 57.29 | 5574748 |
