# Supplementary material for: Within-Host Genotypic and Phenotypic Diversity of Contemporaneous Carbapenem-Resistant Klebsiella pneumoniae from Blood Cultures of Patients with Bacteremia
Source: mBio. 2022 Nov 29;13(6):e02906-22. doi: 10.1128/mbio.02906-22 (PMC9765435; doi:10.1128/mbio.02906-22)
Supplement: TABLE S2 [file mbio.02906-22-s0005.docx]

**Supplemental Table 2. Capsular gene mutations in carbapenem-resistant *Klebsiella pneumoniae***

**ST258-wzi154 (KL107) strains.**

| **ID** | **K locus 107 (wzi154)** | | | | | | | | | | | | | | | |
| --- | --- | --- | --- | --- | --- | --- | --- | --- | --- | --- | --- | --- | --- | --- | --- | --- |
|  | ***galF*** | ***cpsACP*** | ***wzi*** | ***wza*** | ***wzb*** | ***wzc*** | ***wbaP*** | ***fbN*** | ***rfbF*** | **GT** | ***gnd*** | ***rmlB*** | ***rmlA*** | ***rmlD*** | ***rmlC*** | ***ugd*** |
| A1 | + | + | + | + | + | + | + | + | + | + | + | + | + | + | + | + |
| A2 | + | + | + | + | + | + | + | + | + | + | + | + | + | + | + | + |
| A3 | + | + | + | + | + | + | + | + | + | + | + | + | + | + | + | + |
| A4 | + | + | + | + | + | M | + | + | + | + | + | + | + | + | + | + |
| A5 | + | + | + | + | + | + | + | + | + | + | + | + | + | + | + | + |
| A6 | + | + | + | + | + | + | + | + | + | + | + | + | + | + | + | + |
| A7 | + | + | + | + | + | + | + | + | + | + | + | + | + | + | + | + |
| A8 | + | + | + | + | + | + | + | + | + | + | + | + | + | + | + | + |
| A9 | + | + | + | + | + | + | + | + | + | + | + | + | + | + | + | + |
| A10 | + | + | + | + | + | + | + | + | + | + | + | + | + | + | + | + |
| D1 | - | + | + | + | + | + | + | + | + | + | + | + | + | + | + | + |
| D2 | - | + | + | + | + | + | + | + | + | + | + | + | + | + | + | + |
| D3 | - | + | + | + | + | + | + | + | + | + | + | + | + | + | + | + |
| D4 | - | + | + | + | + | + | + | + | + | + | + | + | + | + | + | + |
| D5 | - | + | + | + | + | + | + | + | + | + | + | + | + | + | + | + |
| D6 | - | + | + | + | + | + | + | + | + | + | + | + | + | + | + | + |
| D7 | - | + | + | + | + | + | + | + | + | + | + | + | + | + | + | + |
| D8 | - | + | + | + | + | + | + | + | + | + | + | + | + | + | + | + |
| D9 | - | + | + | + | + | + | + | + | + | + | + | + | + | + | + | + |
| D10 | - | + | - | - | - | + | + | + | + | + | + | + | + | + | + | + |
| F1 | - | + | + | + | + | M | + | + | + | + | + | + | + | + | + | + |
| F2 | - | + | + | + | + | M | + | + | + | + | + | + | + | + | + | + |
| F3 | - | + | + | + | + | M | + | + | + | + | + | + | + | + | + | + |
| F4 | - | + | + | + | + | M | + | + | + | + | + | + | + | + | + | + |
| F5 | - | + | + | + | + | M | + | + | + | + | + | + | + | + | + | + |
| F6 | - | + | + | + | + | M | + | + | + | + | + | + | + | + | + | + |
| F7 | - | + | + | + | + | M | + | + | + | + | + | + | + | + | + | + |
| F8 | - | + | + | + | + | M | + | + | + | + | + | + | + | + | + | + |
| F9 | - | + | + | + | + | M | + | + | + | + | + | + | + | + | + | + |
| F10 | - | + | + | + | + | M | + | + | + | + | + | + | + | + | + | + |
| J1 | - | + | + | + | + | + | + | + | + | + | + | + | + | + | + | + |
| J2 | - | + | - | - | - | - | - | - | - | - | + | + | + | + | + | + |
| J3 | - | + | + | + | + | + | + | + | + | + | + | + | + | + | + | + |
| J4 | - | + | + | + | + | M | + | + | + | + | + | + | + | + | + | + |
| J5 | - | + | - | - | - | + | + | + | + | + | + | + | + | + | + | + |
| J6 | - | + | - | - | - | - | + | + | + | + | + | + | + | + | + | + |
| J7 | - | + | - | - | + | + | + | + | + | + | + | + | + | + | + | + |
| J8 | - | + | + | + | + | M | + | + | + | + | + | + | + | + | + | + |
| J9 | - | + | - | - | - | - | - | - | - | - | + | + | + | + | + | + |
| J10 | - | + | + | + | + | + | + | + | + | + | + | + | + | + | + | + |

ID: Strain identification; cps: capsular polysaccharide; WT: wild-type

M: *wzc* mutation, as follows: Strain A4 (Proline605Glutamine); Strains F1-F10 (Tyrosine710frameshift); Strains J4, J8: Phenylalanine269Leucine

+: presence of a specific capsular gene

–: absence of a specific capsular gene
