# Supplementary material for: Within-Host Genotypic and Phenotypic Diversity of Contemporaneous Carbapenem-Resistant Klebsiella pneumoniae from Blood Cultures of Patients with Bacteremia
Source: mBio. 2022 Nov 29;13(6):e02906-22. doi: 10.1128/mbio.02906-22 (PMC9765435; doi:10.1128/mbio.02906-22)
Supplement: TABLE S4 [file mbio.02906-22-s0007.docx]

**Supplemental Table 4. Within-host, hybrid short- and long-read whole genome sequence comparisons of carbapenem-resistant *Klebsiella pneumoniae* strains from three patients (A G, J).**

| **Strain** | **Contig** | **Length (bp)** | **Plasmid Replicons** | **Resistance genes** | **Virulence genes** |
| --- | --- | --- | --- | --- | --- |
| **A1** | 1 (Chr) | 5,499,994 |  | *blaSHV-158, fosA6, oqxA, oqxB* | *acrAB, ecpABCDER, entABCEFS, fepABCDG, fimABCDEFGHIK, mrkCDFJIH, rpoS, rcsB, galF, gndA, ugd, rfbABD, clbABCDEFGHIJKLMNOPQS, fyuA/psn, irp1, irp2, rcsA, iroE, ybtAETUPQXS, tssBCDFGHJKLM, iutA, ompA, fur, fes, wzi,* KP1_RS17220, KP1_RS17225, KP1_RS17230, KP1_RS17240, KP1_RS17355 |
|  | **2 (Plasmid)** | **166,337** | **IncFIC(FII), IncFIB(AP001918)** | ***tet(A)*** | ***iroBCDEN, iutA, iucABCD*** |
|  | 3 (Plasmid) | 161,666 | FII(pBK30683) | *blaKPC-3, blaTEM-150, blaOXA-9, blaSHV-158, dfrA14, sul2, aph(3'')-Ib, aph(6)-Id, aadA1, aac(6')-Ib-AKT* |  |
| **A4** | 1 (Chr) | 5,498,521 |  | *blaSHV-158, fosA6, oqxA, oqxB* | *acrAB, ecpABCDER, entABCEFS, fepABCDG, fimABCEFGHIK, mrkCDFJIH, rpoS, rcsB, galF, gndA, ugd, rfbABD, clbABCDEFGHIJKLMNOPQS, fyuA/psn, irp1, irp2, rcsA, iroE, ybtAETUPQXS, tssBCDFGHJKLM, iutA, ompA, fur, fes, wzi, KP1_RS17220, KP1_RS17225, KP1_RS17230, KP1_RS17240, KP1_RS17355* |
|  | 2 (Plasmid) | 161,665 | FII(pBK30683) | *blaKPC-3, blaTEM-150, blaOXA-9, blaSHV-158, dfrA14, sul2, aph(3'')-Ib, aph(6)-Id, aadA1, aac(6')-Ib-AKT* |  |
| **G1** | 1 (Chr) | 5,550,531 |  | *blaSHV-158, fosA6, oqxA, oqxB,* ***sul1, aadA2*** | *acrAB, ecpABCDER, entABCEFS, fepABCDG, fimABCDEFGHIK, mrkABCDFJI, rpoS, rcsB, galF, gndA, ugd, rfbABD, clbABCDEFGHIJKLMNOPQS, fyuA/psn, irp1, irp2, rcsA, iroE, ybtAETUPQXS, tssBCDFGHJKLM, iutA, ompA, fur, fes, wzi, KP1_RS17220, KP1_RS17225, KP1_RS17230, KP1_RS17240, KP1_RS17355* |
|  | **2 (Plasmid)** | **167,851** | **IncFII(pKP91), IncFIB(K)** | ***blaKPC-2*** |  |
|  | 3 (Plasmid) | 126,321 | IncR, IncFIB(pQil) | *aac(6')-Ib-AKT, aadA1, aadA2, cmlA1* |  |
|  | 4 (Plasmid) | 9,294 | ColRNAI |  |  |
| **G7** | 1 (Chr) | 5,542,117 |  | *blaSHV-158, fosA6, oqxA, oqxB* | *acrAB, ecpABCDER, entABCEFS, fepABCDG, fimABCDEFGHIK, mrkABCDFJI, rpoS, rcsB, galF, gndA, ugd, rfbABD, clbABCDEFGHIJKLMNOPQS, fyuA/psn, irp1, irp2, rcsA, iroE, ybtAETUPQXS, tssBCDFGHJKLM, iutA, ompA, fur, fes, wzi, KP1_RS17220, KP1_RS17225, KP1_RS17230, KP1_RS17240, KP1_RS17355* |
|  | 2 (Plasmid) | 126,542 | IncR, IncFIB(pQil) | *aac(6')-Ib-AKT, aadA1, aadA2, cmlA1* |  |
|  | 3 (Plasmid) | 9,294 | ColRNAI |  |  |
| **J1** | 1 (Chr) | 5,389,279 |  | *blaSHV-158, fosA6, oqxA, oqxB* | *acrAB, ecpABCDER, entABCEFS, fepABCDG, fimABCDEFGHIK, mrkCDFJIH, rpoS, rcsB, gndA, ugd, rfbA, clbABCDEFGHIJKLMNOPQS, fyuA/psn, irp1, irp2, rcsA, iroE, ybtAETUPQXS, tssBCDFGHJKLM, iutA, ompA, fur, fes, wzi, KP1_RS17355* |
|  | 2 (Plasmid) | 165,212 | FII(pBK30683) | *blaKPC-3, blaTEM-150, blaOXA-9, blaSHV-158, dfrA14, sul2, aph(3'')-Ib, aph(6)-Id, aadA1, aac(6')-Ib-AKT* |  |
|  | 3 (Chr) | 93,071 |  |  |  |
| **J2** | 1 (Chr) | 5,377,661 |  | *blaSHV-158, fosA6, oqxA, oqxB* | *acrAB, ecpABCDER, entABCEFS, fepABCDG, fimABCDEFGHIK, mrkCDFJIH, rpoS, rcsB, gndA, ugd, rfbA, clbABCDEFGHIJKLMNOPQS, fyuA/psn, irp1, irp2, rcsA, iroE, ybtAETUPQXS, tssBCDFGHJKLM, iutA, ompA, fur, fes, KP1_RS17355* |
|  | 2 (Plasmid) | 165,212 | FII(pBK30683) | *blaKPC-3, blaTEM-150, blaOXA-9, blaSHV-158, dfrA14, sul2, aph(3'')-Ib, aph(6)-Id, aadA1, aac(6')-Ib-AKT* |  |
|  | 3 (Chr) | 93,071 |  |  |  |
|  | **4 (Plasmid)** | **4,097** |  |  |  |

Data were generated using hybrid assemblies of long- and short-read WGSs. Plasmid information was mined using long-read WGS. Within-host differences in content are highlighted as bolded text.

Chr: chromosome; bp: basepairs

KP1_RS17220: glycosyltransferase, LPS; KP1_RS17225: glycosyltransferase family 4 protein, LPS; KP1_RS17230: glycosyltransferase, LPS; KP1_RS17240: DUF4422 domain-containing protein, LPS; KP1_RS17355: phosphatase PAP2 family protein, capsule
