# Supplementary material for: Within-Host Genotypic and Phenotypic Diversity of Contemporaneous Carbapenem-Resistant Klebsiella pneumoniae from Blood Cultures of Patients with Bacteremia
Source: mBio. 2022 Nov 29;13(6):e02906-22. doi: 10.1128/mbio.02906-22 (PMC9765435; doi:10.1128/mbio.02906-22)
Supplement: TABLE S5 [file mbio.02906-22-s0008.docx]

**Supplemental Table 5. Core genome single nucleotide polymorphisms and insertion-deletions in carbapenem-resistant *Klebsiella pneumoniae* strains from patients A (A1 and A4) and G (G1 and G4).**

|  | **Position** | **Type** | **A1** | **A4** | **Effect** | **Gene** | | **Product** |
| --- | --- | --- | --- | --- | --- | --- | --- | --- |
| **non-synonymous** | 45741 | snp | A | T | 590A>T (Gln197Leu) | | *phnR* | Putative transcriptional regulator of 2-aminoethylphosphonate degradation operons |
|  | 844117 | snp | C | T | 287G>A (Gly96Asp) | | *fimH* | Type 1 fimbrin D-mannose specific adhesin |
|  | 1262114 | snp | T | G | 229T>G (Tyr77Asp) | | *rseB* | Sigma-E factor regulatory protein RseB |
|  | 1567657 | snp | C | T | 1283G>A (Arg428His) | | *glpA* | Anaerobic glycerol-3-phosphate dehydrogenase subunit A |
|  | 1588622 | snp | C | T | 586C>T (Gln196 stop gained) | | *ompK36* | Outer membrane porin ompK36 |
|  | 1744504 | snp | A | C | 1814A>C (Gln605Pro) | | *wzc* | Putative tyrosine-protein kinase in cps region |
|  | 1971516 | snp | G | T | 982C>A (Pro328Thr) | |  | Hypothetical protein |
|  | 2228874 | del | TG | T | 1210delC (Gln404 frameshift) | | *narX* | Nitrate/nitrite sensor protein NarX |
|  | 2232548 | snp | A | T | 359A>T (Lys120Met) | | *narG* | Respiratory nitrate reductase 1 alpha chain |
|  | 2417503 | snp | A | T | 89T>A (Leu30Gln) | | *codAch2* | Pterin deaminase |
|  | 3030270 | snp | A | G | 658T>C (Cys220Arg) | | *metI_3* | D-methionine transport system permease protein MetI |
|  | 4181531 | snp | C | G | 496C>G (Leu166Val) | | *rcnA* | Nickel/cobalt efflux system RcnA |
|  | 4443485 | snp | C | T | 1325G>A (Arg442Gln) | | *dgt* | Deoxyguanosinetriphosphate triphosphohydrolase |
|  | 4475399 | snp | C | T | 631C>T (Arg211Trp) | | *outN* | Type II secretion system protein N |
|  | 4540957 | snp | T | C | 848A>G (His283Arg) | | *ftsW* | Putative peptidoglycan glycosyltransferase FtsW |
|  | 4686672 | snp | G | A | 28G>A (Asp10Asn) | | *fhuF* | Ferric iron reductase protein FhuF |
| **synonymous** | 49745 | snp | T | C | 312T>C | | *phnV_2* | Putative 2-aminoethylphosphonate transport system permease PhnV |
|  | 907600 | snp | C | T | 402C>T | | *thyA* | Thymidylate synthase |
|  | 1405990 | snp | C | T | 909G>A | | *dapE* | Succinyl-diaminopimelate desuccinylase |
|  | 1596573 | snp | G | A | 21C>T | | *dauA_1* | C4-dicarboxylic acid transporter DauA |
|  | 2705077 | snp | T | C | 1170T>C | | *yjhB* | Putative metabolite transport protein YjhB |
|  | 2790563 | snp | G | A | 687G>A | | *pobB* | Phenoxybenzoate dioxygenase subunit beta |
|  | 3047092 | snp | A | G | 1077T>C | |  | Hypothetical protein |
|  | 3687400 | snp | G | A | 288C>T | | *aptA_1* | Apulose-4-phosphate transketolase subunit A |
|  | 4156596 | snp | C | T | 363G>A | | *mscK* | Mechanosensitive channel MscK |
| **intergenic** | 42479 | del | TG | T |  | |  |  |
|  | 76006 | snp | C | T |  | |  |  |

| **Variant** | **Position** | **Type** | **G1** | **G7** | **Effect** | **Gene** | **Product** |
| --- | --- | --- | --- | --- | --- | --- | --- |
| **non-synonymous** | 3027705 | snp | A | G | 14T>C (Ile5Thr) |  | Hypothetical protein |
|  | 4303499 | snp | C | A | 1622G>T (Gly541Val) | *lon_2* | Lon protease |
|  | 4418723 | snp | A | T | 374T>A (Ile125Asn) | *ecpA_2* | Common pilus major fimbrillin subunit EcpA |
| **synonymous** | 4781213 | snp | G | C | 153C>G | *deoC_2* | Deoxyribose-phosphate aldolase |
| **intergenic** | 1229978 | ins | T | TAAACTAATGGTTCCGCTAACTCGTG |  |  |  |
|  | 1410052 | snp | A | C |  |  |  |
|  | 4358613 | del | AT | A |  |  |  |
|  | 4940499 | snp | A | T |  |  |  |
|  | 4940526 | snp | T | A |  |  |  |

Data were generated using hybrid assemblies of long- and short-read WGSs. *K. pneumoniae* 30660/NJST258_1 served as reference genome. Note that there were no core genome variants observed in strains J1 or J2.

SNP: single nucleotide polymorphim; ins: insertion; del: deletion; Gln: glutamine; Leu: leucie; Gly: glycine; Asp: aspartic acid; Tyr: tyrosine; Arg: arginine; His: histidine; Pro: proline; Thr: threonine; Lys: lysine; Cys: cysteine; Val: valine; Trp: tryptophan; Asn: asparagine; Ile: isoleucine
