# Supplementary material for: Within-Host Genotypic and Phenotypic Diversity of Contemporaneous Carbapenem-Resistant Klebsiella pneumoniae from Blood Cultures of Patients with Bacteremia
Source: mBio. 2022 Nov 29;13(6):e02906-22. doi: 10.1128/mbio.02906-22 (PMC9765435; doi:10.1128/mbio.02906-22)
Supplement: FIG S1 [file mbio.02906-22-s0001.pdf]

Supplemental Figure 1. *ompk36* sequences in carbapenem-resistant *Klebsiella pneumoniae* strains from patient G (G3, G4, G6).

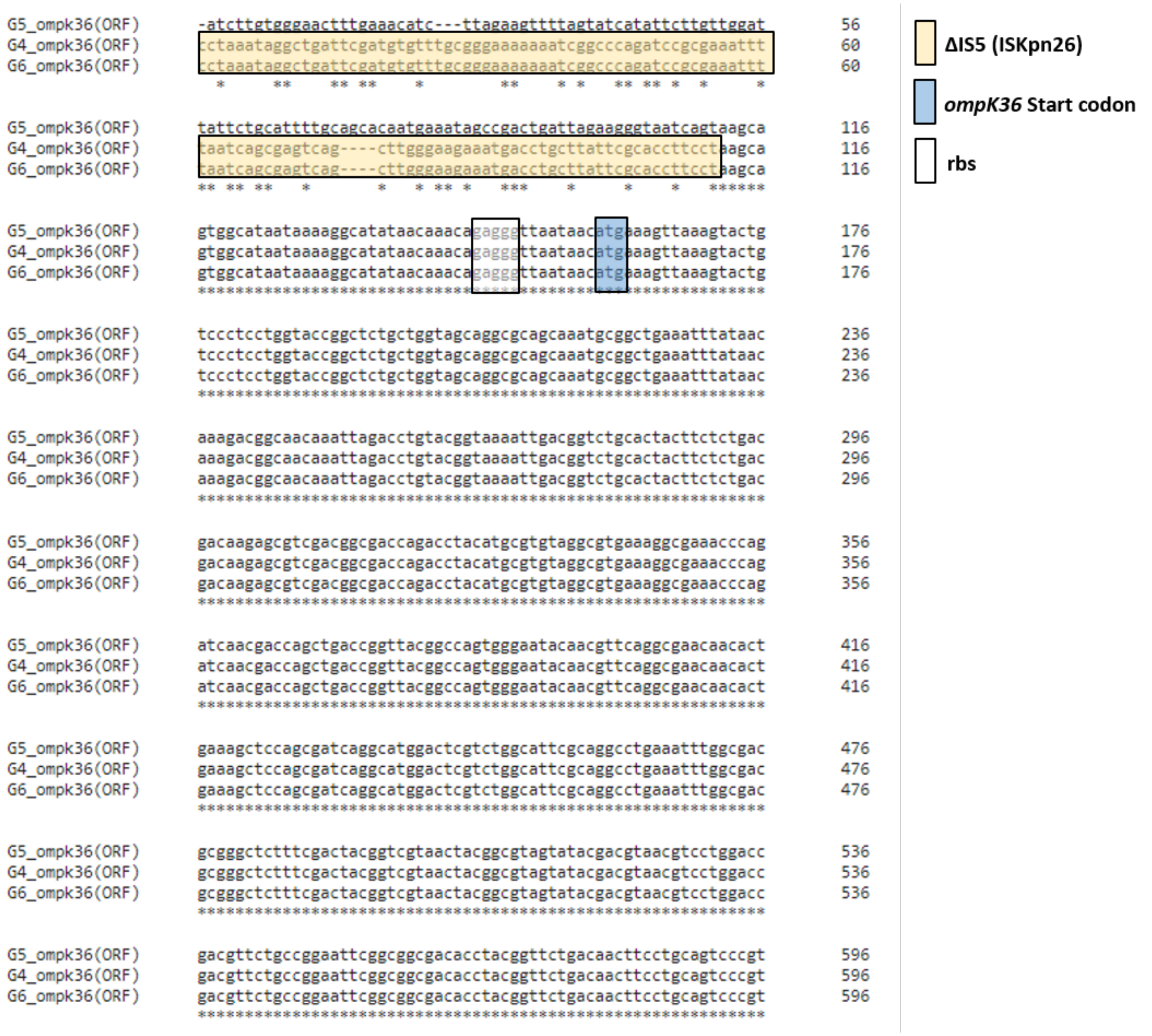

Strains G4 and G6 carried mutations in the promoter region of *ompk36*. *ompk36* sequence in strain G3 was similar to that of the remaining G strains.

rbs: ribosome binding site
