# Supplementary material for: Within-Host Genotypic and Phenotypic Diversity of Contemporaneous Carbapenem-Resistant Klebsiella pneumoniae from Blood Cultures of Patients with Bacteremia
Source: mBio. 2022 Nov 29;13(6):e02906-22. doi: 10.1128/mbio.02906-22 (PMC9765435; doi:10.1128/mbio.02906-22)
Supplement: TABLE S6 [file mbio.02906-22-s0009.docx]

**Supplemental Table 6. Antibiotic minimum inhibitory concentrations against carbapenem-resistant *Klebsiella pneumoniae* strains from three patients (A, G, J).**

| **Strains** | **MEM** | **MVB** | **CAZ** | **CZA** | **TET** | **GEN** |
| --- | --- | --- | --- | --- | --- | --- |
| A1 | >16 | 2 | 128 | 1 | 256 | 4 |
| A2 | >16 | 2 | 64 | 2 | 256 | 2 |
| A3 | >16 | 2 | 128 | 2 | 256 | 4 |
| A4 | >16 | 2 | 64 | 0.5 | **4** | 4 |
| A5 | >16 | 2 | 128 | 2 | 256 | 2 |
| A6 | >16 | 2 | 128 | 1 | 256 | 4 |
| A7 | >16 | 2 | 128 | 0.5 | 256 | 2 |
| A8 | >16 | 2 | 128 | 1 | 256 | 2 |
| A9 | >16 | 2 | 128 | 2 | 256 | 2 |
| A10 | >16 | 2 | 128 | 1 | 256 | 2 |
|  |  |  |  |  |  |  |
| G1 | >16 | 0.06 | 64 | 1 | 4 | 4 |
| G2 | >16 | 0.06 | 64 | 1 | 4 | 4 |
| G3 | >16 | 0.06 | 64 | 1 | 4 | 2 |
| G4 | >16 | **0.25** | 64 | 2 | 4 | 2 |
| G5 | >16 | 0.06 | 128 | 0.5 | 4 | 2 |
| G6 | >16 | **2** | 128 | 4 | 8 | 2 |
| G7 | **0.5** | 0.06 | **0.5** | 0.5 | 4 | 4 |
| G8 | >16 | 0.06 | 64 | 2 | 4 | 2 |
| G9 | >16 | 0.06 | 64 | 1 | 4 | 4 |
| G10 | >16 | 0.06 | 128 | 0.5 | 4 | 4 |
|  |  |  |  |  |  |  |
| J1 | >16 | 0.06 | 128 | 1 | 0.25 | 2 |
| J2 | >16 | 0.06 | 128 | 2 | 0.25 | 2 |
| J3 | >16 | 0.06 | 128 | 1 | 0.25 | 4 |
| J4 | >16 | 0.06 | 128 | 2 | 0.25 | 4 |
| J5 | >16 | 0.06 | 128 | 2 | 0.25 | 4 |
| J6 | >16 | 0.06 | 128 | 1 | 0.25 | 4 |
| J7 | >16 | 0.06 | 128 | 1 | 0.25 | 4 |
| J8 | >16 | 0.06 | 128 | 1 | 0.25 | 4 |
| J9 | >16 | 0.06 | 128 | 1 | 0.25 | 4 |
| J10 | >16 | 0.06 | 128 | 1 | 0.25 | 4 |

Susceptibility testing was performed using the Clinical and Laboratory Standards Institute reference broth microdilution method. Bolded MICs within shaded boxes differ by ≥4-fold from MICs against other strains.

MIC: minimum inhibitory concentration; MEM: meropenem; MVB: meropenem-vaborbactam; CAZ: ceftazidime; CZA: ceftazidime-avibactam; TET: tetracycline; GEN: Gentamycin
