# Supplementary figures and images for: Within-Host Genotypic and Phenotypic Diversity of Contemporaneous Carbapenem-Resistant Klebsiella pneumoniae from Blood Cultures of Patients with Bacteremia
Source: mBio. 2022 Nov 29;13(6):e02906-22. doi: 10.1128/mbio.02906-22 (PMC9765435; doi:10.1128/mbio.02906-22)

**Isolate**

**kDa**

**Ladder**

**G1**

**G4**

**G6**

**G7**

80 →

58 →

46 →

**OmpK36** →

32 →

25 →

22 →

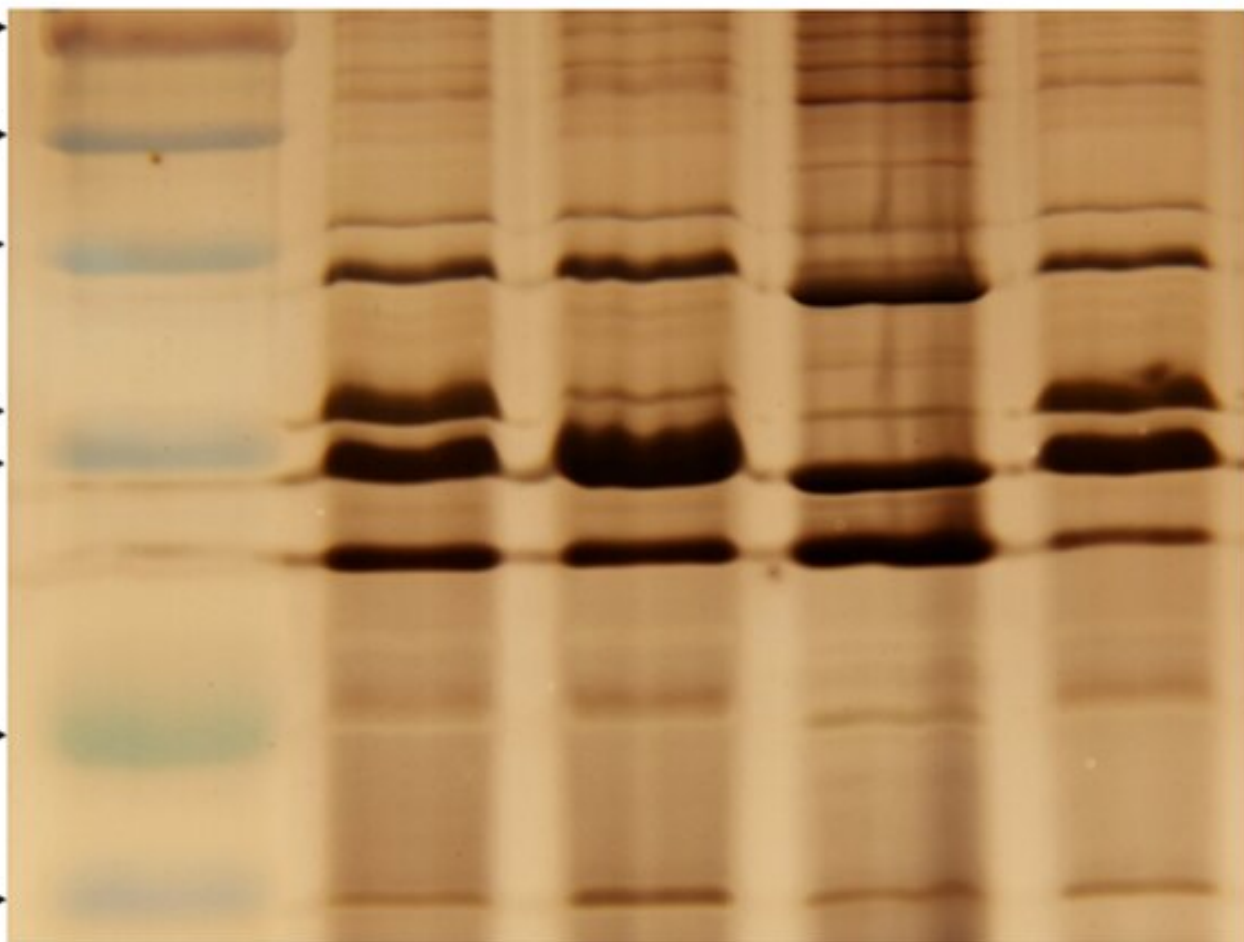

Supplement: FIG S2 [file mbio.02906-22-s0002.pdf]

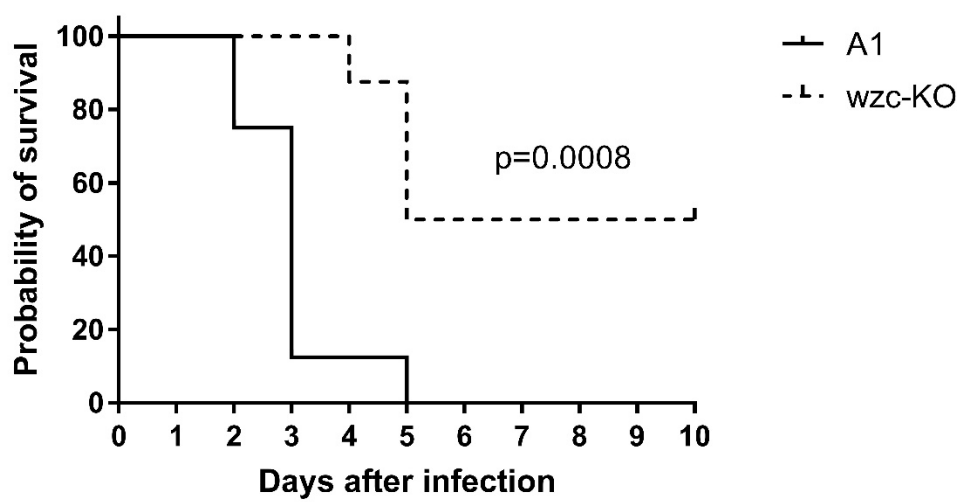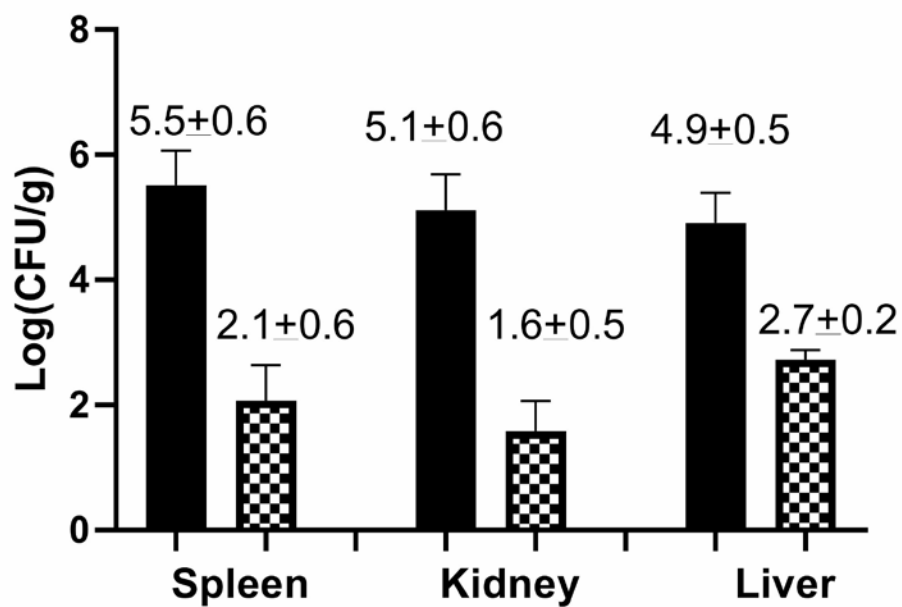

Supplement: FIG S3 [file mbio.02906-22-s0003.pdf]
